# Supplementary material for: CYLD Limits Lys63- and Met1-Linked Ubiquitin at Receptor Complexes to Regulate Innate Immune Signaling
Source: Cell Rep. 2016 Mar 17;14(12):2846–58. doi: 10.1016/j.celrep.2016.02.062 (PMC4819907; doi:10.1016/j.celrep.2016.02.062)

Cell Reports, Volume *14*

Supplemental Information

CYLD Limits Lys63- and Met1-Linked Ubiquitin at Receptor Complexes to Regulate Innate Immune Signaling

Matous Hrdinka, Berthe Katrine Fiil, Mattia Zucca, Derek Leske, Katrin Bagola, Monica Yabal, Paul R. Elliott, Rune Busk Damgaard, David Komander, Philipp J. Jost, and Mads Gyrd-Hansen

Supplemental Information

Supplemental Experimental Procedures

Plasmids and cloning

The plasmids used in this study were obtained from the following sources or generated as described bellow: pcDNA3-HOIP^{WT}-V5/His, pcDNA3-HOIP^{mutRx2} -V5/His, and pcDNA3-HOIL1-V5/His were a kind gift from Prof. Henning Walczak (University College London, London, UK). The mutated HOIP constructs pcDNA3-HOIP$^{PUB+NZF}$-V5/His and pcDNA3-HOIP^{N102D}-V5/His were described previously (Elliott et al., 2014). The OTULIN constructs pcDNA3-HA-OTULIN^{WT}, pcDNA3-HA-OTULIN^{C129A}, and pcDNA3-3xHA-Ub-AVPI-4xUbGS were described previously (Fiil et al., 2013). The pGEX-6P-1-Ubiquitin4x construct was subcloned from a plasmid kindly provided by Prof. Ivan Dikic (Goethe University, Frankfurt am Main, Germany). The NF-κB luciferase reporter plasmids pBIIX-Luc and TK-renilla-Luc and the XIAP constructs pcDNA3-3xHA-XIAP^{WT}, pcDNA3-3xHA-XIAP^{F495A} have been described previously (Damgaard et al., 2012; Gyrd-Hansen et al., 2008). The pCMV-2-FLAG-CYLD^{WT} was cloned from human cDNA and the catalytically inactive mutant FLAG-CYLD^{C601A} was generated by PCR-based site-directed mutagenesis. The constructs for ubiquitin binding pGEX-6P-1-NEMO-CoZi1-His6 (M1-SUB) and pEGFP-C1-NEMO-CoZi (GFP-M1-SUB) and pGEX-6P-1-Ubiquillin-UBA4x (TUBE) were described previously (Fiil et al., 2013). The pGEX-6P-1-Ubiquillin-UBA1x construct consisting of a single UBA domain was generated by PCR. The constructs for Lys63-linked polyubiquitin binder pET104-DEST-RAP80-UIM3x (K63-SUB) was a kind gift from Niels Mailand and Tina Thorslund and was cloned into pcDNA-DEST53-RAP80-UIM3x to make GFP-K63-SUB. To create GFP-K63-SUB, GFP-M1-SUB and GFP alone targeted to the nucleus, the SV40 nuclear localization sequence (NLS, sequence: PKKKRKV) was inserted between GFP and the Ub binder. Full sequences will be made available upon request.

Antibodies and immunoprecipitation reagents

The following antibodies and reagents were used according to the manufacturers' instructions: rat monoclonal anti-HA (Roche Diagnostics, Burgess Hill, UK), mouse monoclonal anti-JNK1/2 and anti-phospho-JNK (Tyr183/Tyr185) (BD Biosciences, San Jose, CA), rabbit monoclonal anti-JNK, ERK1/2 and phospho-ERK1/2 (Cell Signaling Technology, Danvers, MA, Figure S3B), mouse monoclonal anti-

XIAP (Abcam, Cambridge, MA), mouse monoclonal anti-ubiquitin (Imgenex, San Diego, CA, or P4D1, Cell Signaling Technology), rabbit monoclonal Lys63 antibody (Millipore), rabbit monoclonal anti-RIP1 (D94C12), rabbit polyclonal and mouse monoclonal anti-RIPK2 (Santa Cruz Biotechnology, Santa Cruz, CA), mouse monoclonal anti-RIPK2 (Figure S3B, BD Biosciences), rabbit polyclonal and mouse monoclonal anti-NEMO/IKKγ (Santa Cruz Biotechnology and BD Biosciences, respectively), and rabbit polyclonal anti-SHARPIN (ProteinTech, Chicago, IL), rabbit polyclonal anti-RBCK1/HOIL-1 (Novus Biologicals, Littleton, CO). Rabbit polyclonal anti-HOIP/RNF31 (Sigma-Aldrich, Gillingham, UK or Bethyl Laboratories, Montgomery, USA). Mouse monoclonal anti-V5 (AbD Serotec, Kidlington, UK), monoclonal anti-β-actin (Chemicon Millipore, Billerica, MA), rabbit polyclonal antibodies to RelA/p65, p38 MAP Kinase and IκBα, and rabbit polyclonal antibodies to phosphorylated p38 MAP Kinase (Thr180/Tyr182), RelA/p65 (Ser536) and IκBα (Ser32) (Cell Signaling Technology), rabbit polyclonal anti-Fam105B/OTULIN (Abcam), mouse monoclonal and rabbit polyclonal anti-CYLD (Santa Cruz Biotechnology, and Cell Signaling Technology, respectively), rabbit monoclonal anti-A20/TNFAIP3 (D13H3, Cell Signaling Technology), rabbit polyclonal anti-Ubc13 (Cell Signaling Technology), and rat monoclonal anti-cIAP1 (Enzo Life Sciences, Exeter, UK). The HRP-conjugated secondary antibodies were purchased from Bio-Rad (anti-rabbit) and Dako (anti-mouse). For immunofluorescence, primary antibodies rabbit polyclonal anti-RelA (Santa Cruz Biotechnology) or rabbit monoclonal anti-NF-κB p65 (D14E12, Cell Signaling), mouse monoclonal anti-FLAG M2 (Sigma-Aldrich) and secondary antibodies anti-mouse IgG / Alexa Fluor 488 and anti-rabbit IgG/Alexa Fluor 568 (Invitrogen Life Technologies) were used. For intracellular staining of IL-8 for flow cytometry the APC conjugated mouse anti-human IL-8 Antibody (clone E8N1, BioLegend, SanDiego, CA) was used. For immunoprecipitation anti-HA-agarose conjugate (Clone HA-7, A2095), anti-FLAG affinity gel (Clone M2, A2220) and anti-V5 affinity gel (Clone V5-10, A7345) were from Sigma-Aldrich.

Cell lines

NOD2-expressing U2OS-Flp-In™ T-REx™ (U2OS/NOD2) cells were cultured in DMEM GlutaMax (Gibco Life Technologies) supplemented with 10 % (v/v) FBS (Sigma-Aldrich) and 1 % (v/v) Penicillin-Streptomycin (Gibco Life Technologies) and transfected using Fugene 6 (Promega). Throughout the study, the U2OS/NOD2 cells were cultured and stimulated in the absence of doxycycline unless otherwise indicated. HEK293T (Figure 3E, Suppl. Figure S3D) and HEK293FT were cultured in DMEM

GlutaMax (Gibco Life Technologies) supplemented with 10 % (v/v) FBS (Sigma-Aldrich) and 1 % (v/v) Penicillin-Streptomycin (Gibco Life Technologies) and transfected using Fugene HD (Promega). THP1 cells were maintained at density not exceeding $1x10^6$ cells per ml of culture media RPMI1640 GlutaMax (Invitrogen) supplemented with 10 % (v/v) FBS (Sigma-Aldrich), 1 % (v/v) Penicillin-Streptomycin (Gibco Life Technologies), 1 mM sodium pyruvate (Gibco Life Technologies), and 50 μM 2-mercaptoethanol (Lonza). The HCT-116 cells were cultured in McCoy's GlutaMax medium (Gibco Life Technologies) supplemented with 10 % (v/v) FBS (Sigma-Aldrich) and 1 % (v/v) Penicillin-Streptomycin (Gibco Life Technologies).

BMDC generation

For generation of bone marrow derived dendritic cells (BMDC), the bone marrow from WT and *Cyld*$^{-/-}$ mice was cultured in VLE-RPMI-medium (Biochrom Merck Millipore) supplemented with 10 % Sera plus FBS (PAN Biotech), Glutamine, Penicillin-Streptomycin, β-mercapto-ethanol (Gibco), and 20 nM GM-CSF (Peprotech). Culture medium was supplemented on day 3 and day 6 and experiments were performed on day 7. *Cyld*$^{-/-}$ mice were obtained from Prof. Dr. Dirk Schlüter, Otto-von-Guericke-University Magdeburg, Germany.

Luciferase reporter assays

HEK293FT, HCT-116 WT or HCT-116 HOIP KO cells were co-transfected with the NF-κB luciferase reporter construct pBIIX-luc and a thymidine kinase-renilla luciferase construct for normalization of transfection efficiency. HEK293FT and HCT-116 cells were co-transfected with additional plasmids as indicated elsewhere and assays were performed as previously described (Damgaard et al., 2012). Individual experiments were performed in duplicate.

Immunoprecipitation

Immunoprecipitation of HA-NOD2, FLAG-CYLD, endogenous SHARPIN, or HOIP-V5 from U2OS/NOD2 or HEK293T/FT cells. Cells were transfected and treated as indicated. Cells were lysed in the IP buffer (25 mM HEPES pH 7.4, 150 mM KCl, 2 mM $MgCl_2$, 1 mM EGTA, 0.5 % (v/v) Triton X-100) supplemented with 5 mM N-Ethylmaleimide (NEM; Sigma Aldrich), cOmplete protease inhibitor cocktail and PhosSTOP (Roche) for 30 min on ice. Lysates were cleared by centrifugation and were

incubated at 4°C overnight with anti-HA (Sigma Aldrich), anti-FLAG affinity gel (Sigma Aldrich), anti SHARPIN (ProteinTech), or anti-V5-coupled beads (Sigma Aldrich). Beads were washed four times in 500 µl of ice-cold IP buffer and bound material eluted with 0.2 M glycine pH 2.5.

TNF receptor pull-down

U2OS/NOD2 cells were treated with FLAG-TNF (Enzo, 100 ng/ml) for indicated time points. Cells were lysed in buffer containing: 30 mM TrisHCl, 120 mM NaCl, 2 mM EDTA, 2 mM KCl, 1 % Triton X-100 supplemented with 1 mM DTT, 5 mM N-Ethylmaleimide (NEM), cOmplete protease inhibitors, and phosSTOP (Roche). Lysates were incubated with anti-FLAG-agarose beads over night at 4°C. The bound material was eluted with 100 µg/ml FLAG peptide (Sigma Aldrich).

Receptor stimulation

Cells were treated with NOD2 ligand L18-MDP (InvivoGen) or a recombinant TNF receptor ligand TNF-α (Peprotech, Rocky Hill, NJ) added directly to the culture medium for the times indicated. Cells were lysed in TUBE lysis buffer (20 mM Na_2HPO_4, 20 mM NaH_2PO_4, 1 % NP-40, 2 mM EDTA) supplemented with 5 mM N-Ethylmaleimide (NEM; Sigma Aldrich), cOmplete protease inhibitor cocktail and PhosSTOP and cleared by centrifugation before lysis loading sample buffer (LSB) was added. BMDC were stimulated with 10 μg/ml MDP (Invivogen) in the absence of GM-CSF and for BMDCs time course, cells were harvested, washed in PBS and lysed in RIPA buffer supplemented with cOmplete Protease Inhibitor Cocktail (Roche) and the phosphatase inhibitors sodium orthovanadate and sodium flouride (Sigma-Aldrich).

RNA isolation, cDNA synthesis and qPCR

Total RNA was isolated from cells using RNeasy Mini Kit (Qiagen) and DNase digestion was performed on-column with the RNase-Free DNase Set (Qiagen) according to manufacturer's protocol. Total RNA was reverse transcribed with RevertAid™ Reverse Transcriptase (Thermo-Fisher Scientific) and a mixture of anchored oligo(dT)$_{20}$ primers and random pentadecamers in the presence of RiboLock (Thermo-Fisher Scientific). qPCR was performed using SYBR Select Master Mix (Applied Biosystems).

cDNA was amplified with the following primer pairs:

Hypoxanthine phosphoribosyltransferase (HPRT; used as reference gene for normalization):

5'-AGCCAGACTTTGTTGGATTTG-3' and 5'-TTTACTGGCGATGTCAATAGG-3',

TNF: 5'-TGCTGCAGGACTTGAGAAGA-3' and 5'-GAGGAAGGCCTAAGGTCCAC-3',

IL-8: 5'-TCTGGCAACCCTAGTCTGCT-3' and 5'-AAACCAAGGCACAGTGGAAC -3',

OTULIN: 5'-ACATGAAAGAGGGGCATCAG-3' and 5'-TTCATACACGTTGCTTTCTGTGT-3'

CYLD: 5'-TTTGCGTGTGTTGAAAGTACAAT-3' and 5'-TTCCTGCGTCACACTCTCTG-3'

A20: 5'-ATGCACCGATACACACTGGA-3' and 5'-GGATGATCTCCCGAAACTGA-3'

All experiments were performed three times with two technical replicates per experiment.

Transient and stable RNAi knock-down

The cells were reverse transfected with siRNA oligonucleotides (final concentration 35 nM siRNA oligo) using Lipofectamine RNAiMAX (Invitrogen Life Technologies) according to the manufacturer's instructions. The following siRNA oligonucleotides (Sigma-Aldrich) were used for RNAi-mediated knockdown of OTULIN: SASI_Hs01_00040471, CAAAUGAGGCGGAGGAAUA[dT][dT]; CYLD: SASI_Hs02_00309209, GAACAGAUUCCACUCUUUA[dT][dT], Ubc13 #1: SASI_Hs01_00012964, CAGACAUCUUCAGUCCUUU[dT][dT]; Ubc13 #2: SASI_Hs01_00012965, GAAGAAUAUGUUUAGAUAU[dT][dT]; HOIP: GGCGUGGUGUCAAGUUUAA[dT][dT] (Haas et al., 2009); Mismatched control (siMM): Mission siNEG Ctrl 1 (Cat. #SIC001).

To generate stable knock-down of CYLD (shCYLD) and mismatch control knock-down (shMM) in U2OS/NOD2, THP1 and HCT-116 cells the validated siRNA oligos were cloned into pSUPER (BglII/HindIII) vector as 60-bp hairpins. shCYLD: 5'-GATCCCCGAACAGATTCCA CTCTTTATTCAAGAGATAAAGAGTGGAATCTGTTCTTTTTA-3', shMM: 5'-GATCCCCTTCTC CGAACGTGTCACGTTTCAAGAGAACGTGACACGTTCGGAGAATTTTTA-3'

The hairpins were then subcloned into pTRIP-GFP (Watabe-Uchida et al., 2006) or pTRIP-PURO lentiviral vectors via EcoRI site and validated by sequencing. The shRNA vector TRC2-pLKO-PURO for stable OTULIN knock-down (shOTLN) was purchased from Sigma-Aldrich (Cat. #TRCN0000285391). The lentiviral particles were generated in HEK293FT cells by cotransfection of the shRNA vectors with packaging vectors psPAX2 and pMD.G. The virus-containing supernatants were harvested after 72 h, filtered through 0.45 μm filters, and concentrated by precipitation with PEG-

8000. The cells were infected with the concentrated lentiviral particles in the presence of 6-10 μg/ml polybrene (Sigma-Aldrich) over night and selected with 1 μg/ml puromycin for one week.

Expression and purification of ubiquitin binding entities for pull down

E. coli BL21 cells were transfected with plasmids encoding C-terminally His6-tagged ubiquitin binding domains. For the expression and *in vivo* biotinylation of the K63-SUB, BL21 cells expressing the BirA biotin ligase from a pCDF-Duet vector were used. Over night cultures were diluted 1:100 into TB medium supplemented with appropriate antibiotics. Cells were grown at 37°C for 6 h, cooled down to 16°C, protein expression was induced with 500 *μ*M IPTG followed by incubation for 17 h at 16°C and 180 rpm before harvesting. The cells were lysed in His6-binding buffer (50 mM Na-phosphate pH 7.5, 300 mM NaCl, 10 % Glycerol, 10 mM Imidazole, 0.5 mM TCEP) using a high-pressure homogenizer. Lysate was cleared and loaded on a FPLC for protein purification via a 5 ml HisTrap FF column. After injecting the sample, the column was washed with ten column volumes of 50 mM Na-phosphate pH 7.5, 300 mM NaCl, 10 % Glycerol, 30 mM Imidazole, 0.5 mM TCEP followed by protein elution with 50 mM Na-phosphate pH 7.5 , 300 mM NaCl, 10 % Glycerol, 300 or 500 mM Imidazole, 0.5 mM TCEP. Subsequent dialysis of protein solution was performed in 2-3 steps in 50 mM Na-phosphate pH 7.5, 150 mM NaCl, 10 % Glycerol, 0.5 mM TCEP. Proteins were frozen in liquid nitrogen and stored at -80°C.

Expression and purification of GST-Ubiquitin4x

E coli BL21 Rosetta chemically competent cells were transfected with the GST-Ubiquitin4x construct and grown over night in LB, 10 % TB, Ampicillin, shaking at 37°C. Cells were diluted 1:10 in 1 l of LB, 10 % TB, Ampicillin and grown at 37°C until $OD_{(600)}$ = 0.6. Cells were cooled down on ice, protein expression was induced by adding 0.1 mM IPTG and grown over night at 18°C before harvesting. Lysis of cells was performed in PBS with 300 mM NaCl, 1 mM DTT, cOmplete protease inhibitor mix (Roche) by sonication. Lysate was cleared by centrifugation and incubated for 2 h with equilibrated Glutathione Sepharose™ 4 Fast Flow, rotating at 4°C. Resin was washed with TBS before elution of the purified proteins using (50 mM Tris-HCl pH 8.5, 150 mM NaCl, 10 % glycerol, 2 mM β-mercaptoethanol, 20 mM reduced glutathione). Proteins were stored at -80°C in TBS, 10 % Glycerol, and 1 mM DTT.

Supplemental Figure Legends

Figure S1. CYLD antagonizes LUBAC function but does not affect HOIP ubiquitination. Data are associated with Figure 1.

(A) HEK293FT cell lysates from experiment shown in Figure 1D were analyzed by immunoblotting as indicated. (B) *In vitro* DUB assay on Lys63 or GST-Met1 tetraUb with CYLD WT and catalytically inactive (C601A) isolated via their FLAG-tag followed by immunoblotting or coomassie staining to determine level of purified CYLD. (C) Immunoblot for Ub from experiment shown in Figure 1E of purified Ub-conjugates using the UBA^{Ubq}, M1-SUB or K63-SUB from control U2OS/NOD2 cells or cells depleted for CYLD (shCYLD) or OTULIN (shOTLN) as indicated after treatment with L18-MDP (200 ng/ml, 1 h) or TNF (10 ng/ml, 10 min). (D) and (E) Immunoblot for Ub in purified Ub-conjugates using the M1-SUB from THP1 cells (D) from the experiment shown in Figure 1F or HCT-116 cells (E) from the experiment shown in Figure 1G. (F) Purified Ub conjugates using M1-SUB and TUBE from HEK293FT from the experiment shown in Figure 1H were analyzed by immunoblotting as indicated. Asterisk denotes unspecific band detected by the antibody.

Figure S2. CYLD restricts NOD2 signaling and cytokine production. Data are associated with Figure 2.

(A) Data from Figure 2D plotted as mean fluorescence intensity (MFI) of all cells in the IL-8/APC channel. (B) Immunoblotting of key NF-κB and MAPK signaling proteins in U2OS/NOD2 control, OTULIN and/or CYLD-depleted cells upon L18-MDP treatment (5 ng/ml or 200 ng/ml for 1 h and 2 h). (C) NF-κB activity in HCT-116 WT or HOIP KO cell lysates transfected with luciferase reporters and HOIP WT where indicated. Cells were treated for 24 h with L18-MDP (200 ng/ml) or TNF (10 ng/ml) as indicated. To the right, HCT-116 WT and HOIP KO cell lysates were analyzed by immunoblotting as indicated. Data in (A) and (C) represent the mean ±SEM of at least three independent experiments, each performed in duplicate. **$p < 0.01$, *n.s.,* not significant.

Figure S3. CYLD catalytic activity inhibits the NOD2 pathway upstream of nuclear translocation of NF-κB. Data are associated with Figure 3.

(A) Data from Figure 3C plotted as mean fluorescence intensity (MFI) of all cells in the IL-8/APC channel. (B) Untransfected cells from the experiment shown in Figure 3C plotted as percentage of IL-8

positive cells and MFI for all cells in the IL-8/APC channel. (C) and (D) HEK293FT cell lysates from experiments shown in Figure 3D (C), Figure 3E (D) were analyzed by immunoblotting as indicated (F) Schematic depiction of the engineered AVPI-Ub4GS protein binding to XIAP BIR2 and BIR3 domains via an N-terminal IBM to activate NF-κB. (F) NF-κB activity in HEK293FT cell lysates transfected with dual luciferase reporters and vector, inactive $XIAP^{F/A}$, AVPI-Ub4GS, CYLD or OTULIN as indicated, below HEK293FT cell lysates were analyzed by immunoblotting as indicated.

Data in (A), (B) and (F) represent the mean ±SEM of at least three independent experiments, each performed in duplicate (D-F). $^{**}p < 0.01$, *n.s.,* not significant.

Figure S4. CYLD limits extension of Ub chains on RIPK proteins. Data are associated with Figure 4.

(A) Endogenous Ub conjugates from Figure 4A purified using biotin-K63-SUB from U2OS/NOD2 cells and cell lysates at the indicated time points after treatment with L18-MDP (200 ng/ml).

Figure S5. Inhibition of IAPs reveals extensive regulation of RIPK2 ubiquitination by CYLD and OTULIN. Data are associated with Figure 5.

(A) and (B) Purified Ub conjugates using TUBE or UBA^{Ubq} as indicated from U2OS/NOD2 cells from the experiment shown in Figure 5A (A) and Figure 5B (B) were analyzed by immunoblotting against Ub.

Figure S6. CYLD trims Lys63- and Met1-Ub on RIPK2. Data are associated with Figure 6.

(A-C) UbiCRest analysis of ubiquitinated RIPK2 isolated with UBA^{Ubq} from L18-MDP treated U2OS/NOD2 (A and B) and THP-1 (C) cells (200 ng/ml, 1 h). Purified Ub conjugates were incubated with the indicated deubiquitinases with or without a spike in of GST-Met1-Ub4 for the indicated time at 30°C, and samples were examined by immunoblotting of RIPK2 or antibodies that recognize all Ub linkages, Lys63-Ub or Met1-Ub. The anti-RIPK2 blots in (A) are from the same gel but have been cut in two and tilted to adjust for bending of the gel. Asterisk in (B) denotes cross-reactivity of the RIPK2 antibody with recombinant GST-Met1-Ub4 added to the reaction.

Figure S7. Lys63-Ub and Met1-Ub are individually indispensable for NOD2 signaling. Data are associated with Figure 7.

(A) Mean fluorescence intensity of all U2OS/NOD2 cells in the GFP channel transfected with GFP, GFP-K63-SUB or GFP-M1-SUB with or without the nuclear localization signal. The cells were either left untreated or stimulated with L18-MDP (200 ng/ml, 4 h). (B) Data from Figure 7D plotted as mean fluorescence intensity (MFI) of all cells in the IL-8/APC channel. Data represent the mean ±SEM of at least three independent experiments, each performed in duplicate. $^{**}p < 0.01$, $^{***}p < 0.001$.

Supplemental References

Gyrd-Hansen, M., Darding, M., Miasari, M., Santoro, M.M., Zender, L., Xue, W., Tenev, T., da Fonseca, P.C., Zvelebil, M., Bujnicki, J.M., *et al.* (2008). IAPs contain an evolutionarily conserved ubiquitin-binding domain that regulates NF-kappaB as well as cell survival and oncogenesis. Nat Cell Biol *10*, 1309-1317.

Watabe-Uchida, M., John, K.A., Janas, J.A., Newey, S.E., and Van Aelst, L. (2006). The Rac activator DOCK7 regulates neuronal polarity through local phosphorylation of stathmin/Op18. Neuron *51*, 727-739.

Figure S1

A
for Figure 1D

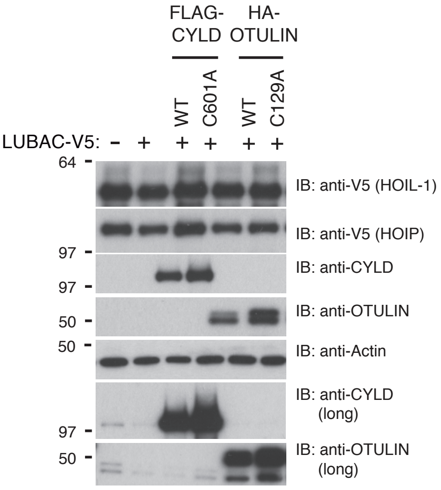

B
For Figure 1D and 1H

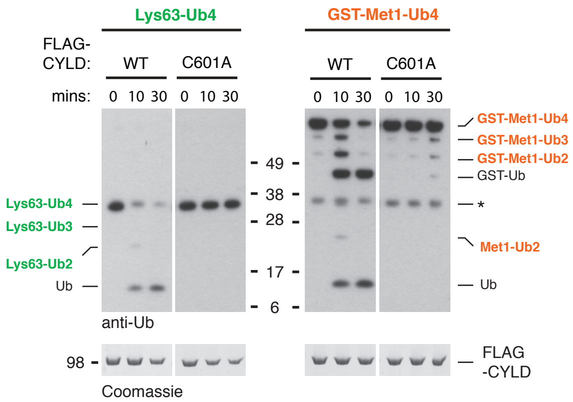

C
for Figure 1E

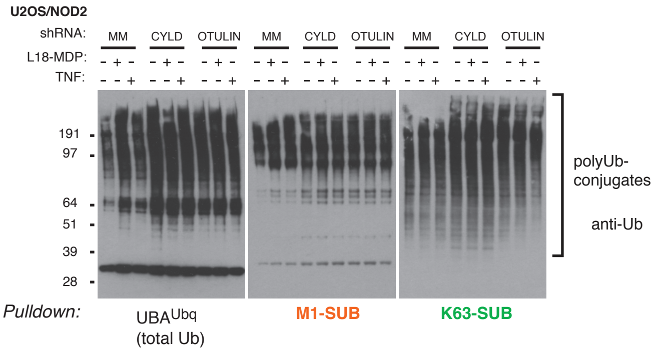

D
for Figure 1F

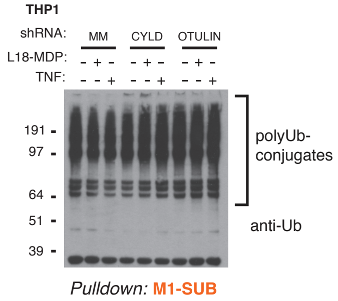

E
for Figure 1G

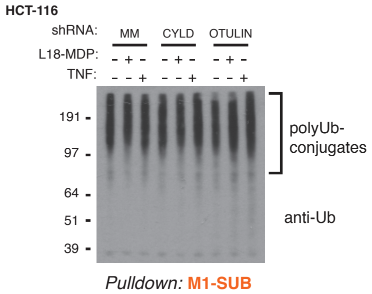

F
for Figure 1H

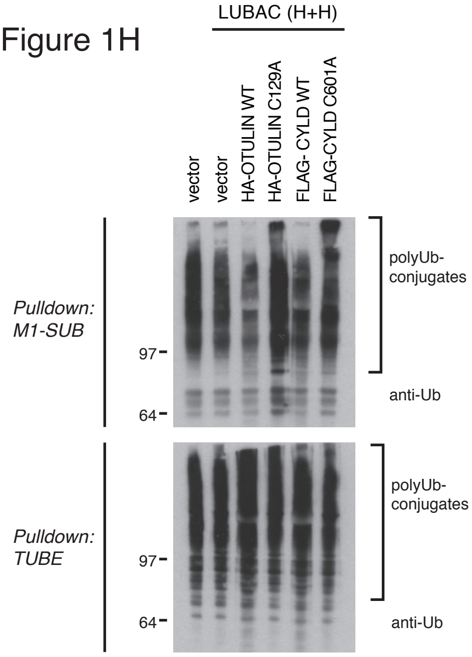

Figure S2

A
for Figure 2D

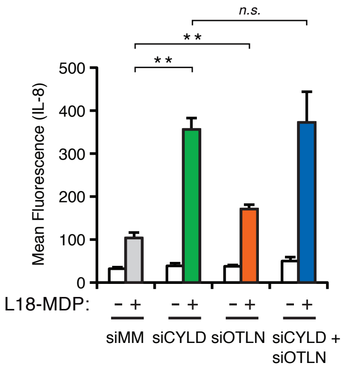

B
for Figure 2D

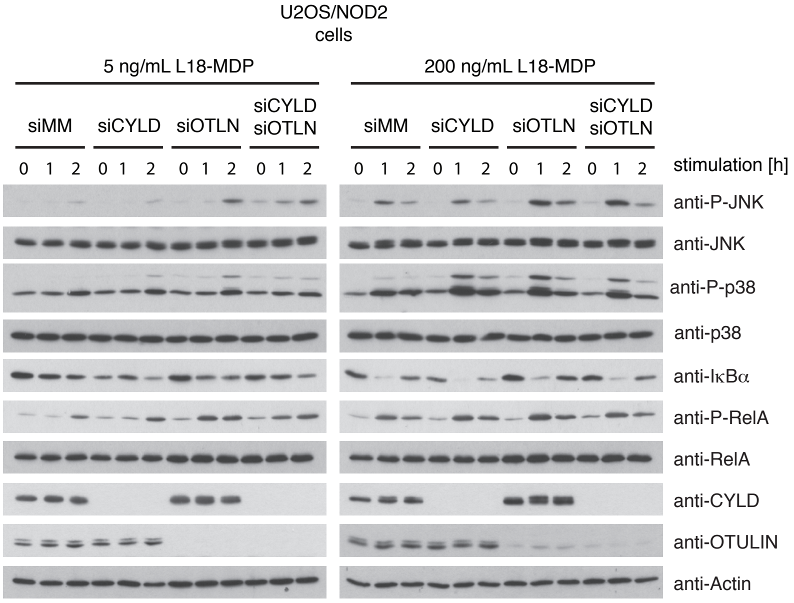

C
for Figure 2F

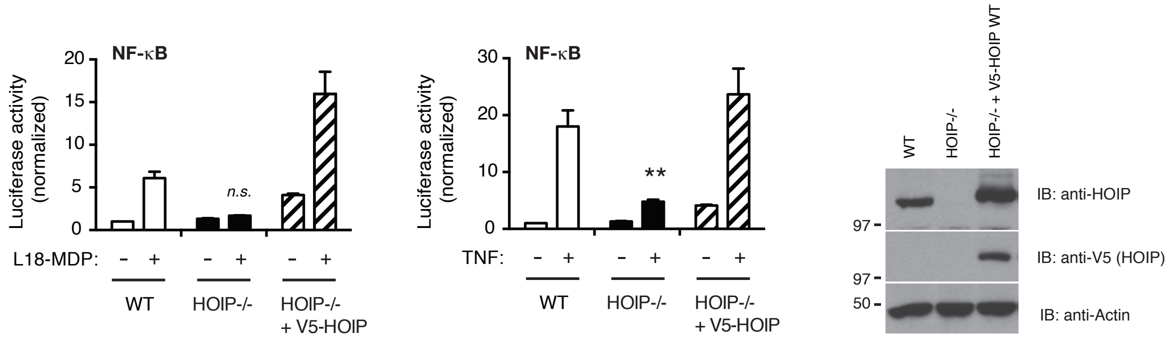

Figure S3

A
for Figure 3C

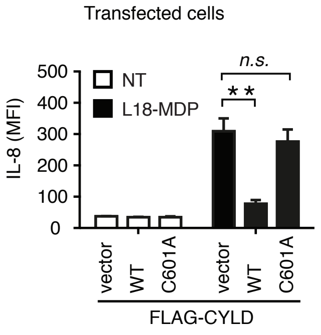

B
for Figure 3C

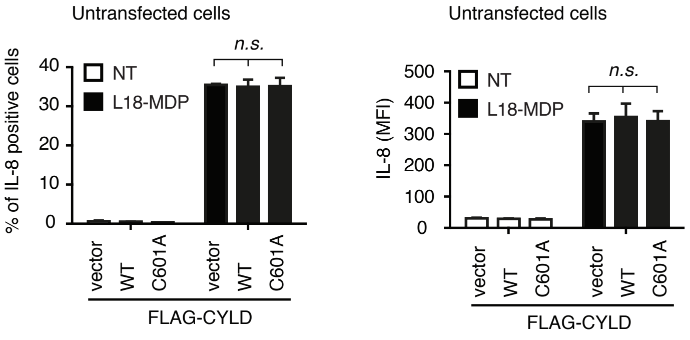

C
for Figure 3D

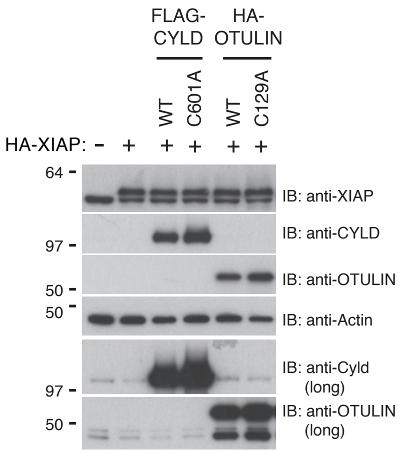

D
for Figure 3E

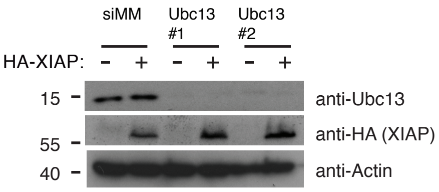

E
for Figure 3D

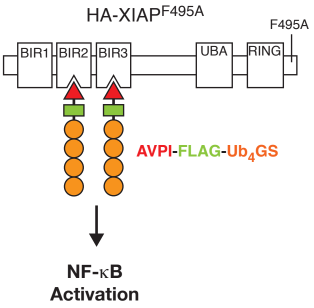

F
for Figure 3D

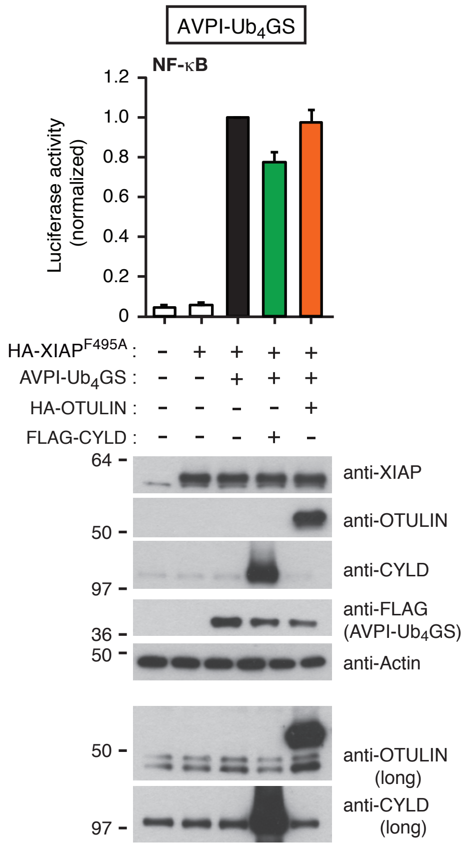

Figure S4

A
for Figure 4A

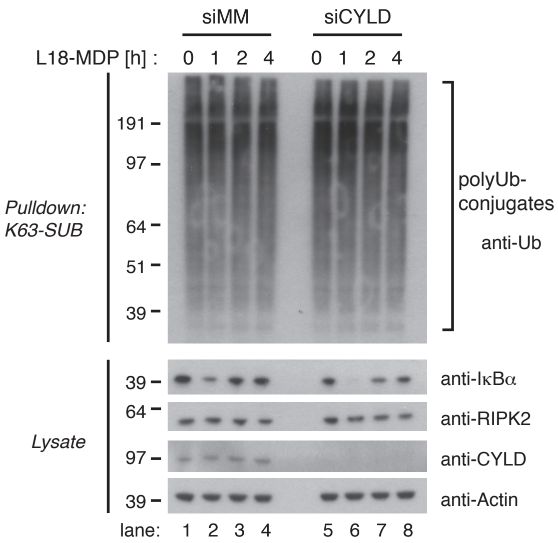

Figure S5

A
for Figure 5A

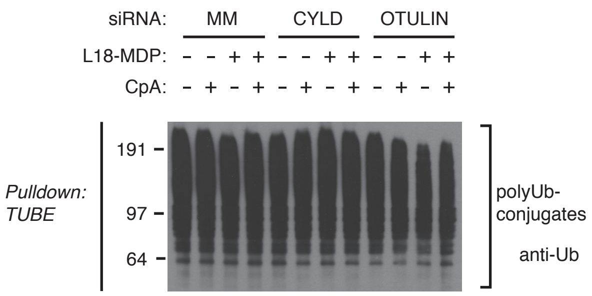

B
for Figure 5B

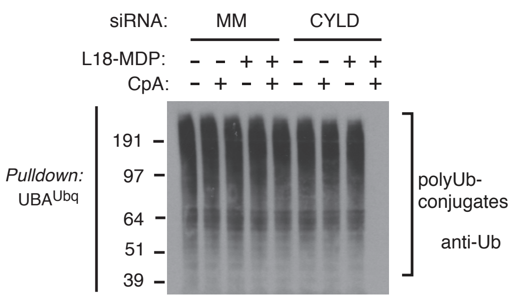

Figure S6

A
for Figure 6B

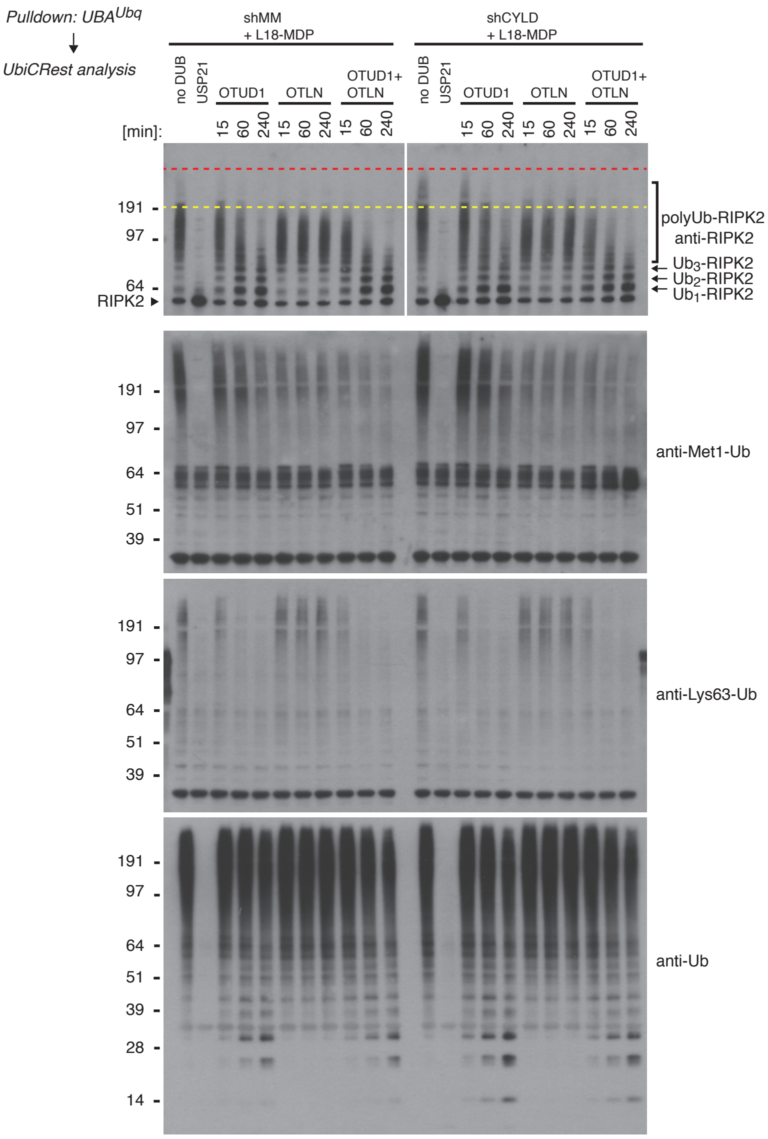

B
for Figure 6B

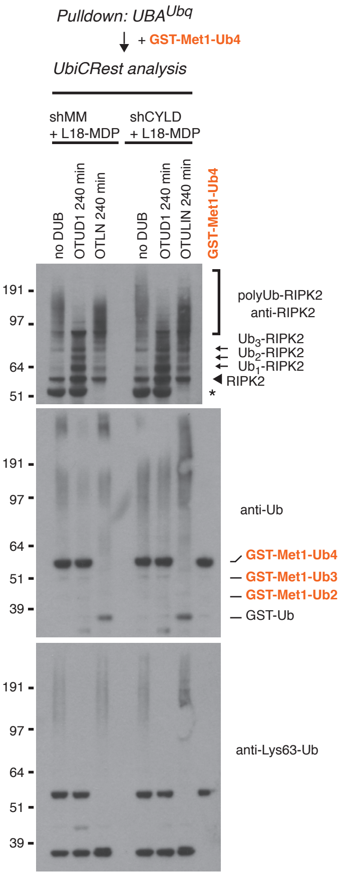

C
for Figure 6C

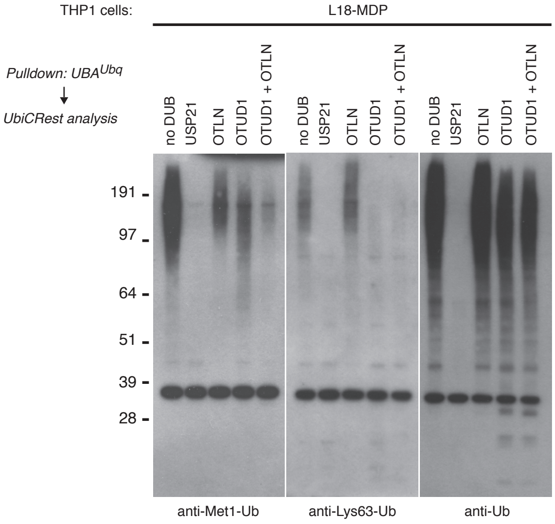

Figure S7

A
for Figure 7D and 7F

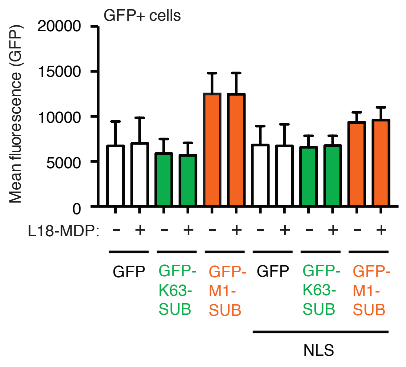

B
for Figure 7D

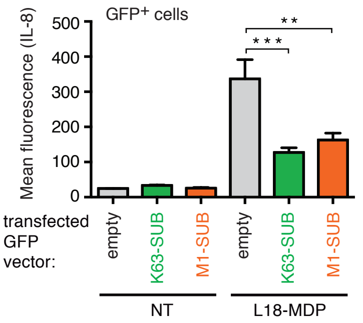

Supplement: Document S1. Supplemental Experimental Procedures and Figures S1–S7 [file mmc1.pdf]
