# Supplementary material for: CYLD Limits Lys63- and Met1-Linked Ubiquitin at Receptor Complexes to Regulate Innate Immune Signaling
Source: Cell Rep. 2016 Mar 17;14(12):2846–58. doi: 10.1016/j.celrep.2016.02.062 (PMC4819907; doi:10.1016/j.celrep.2016.02.062)

Cell Reports

Article

CYLD Limits Lys63- and Met1-Linked Ubiquitin at Receptor Complexes to Regulate Innate Immune Signaling

Graphical Abstract

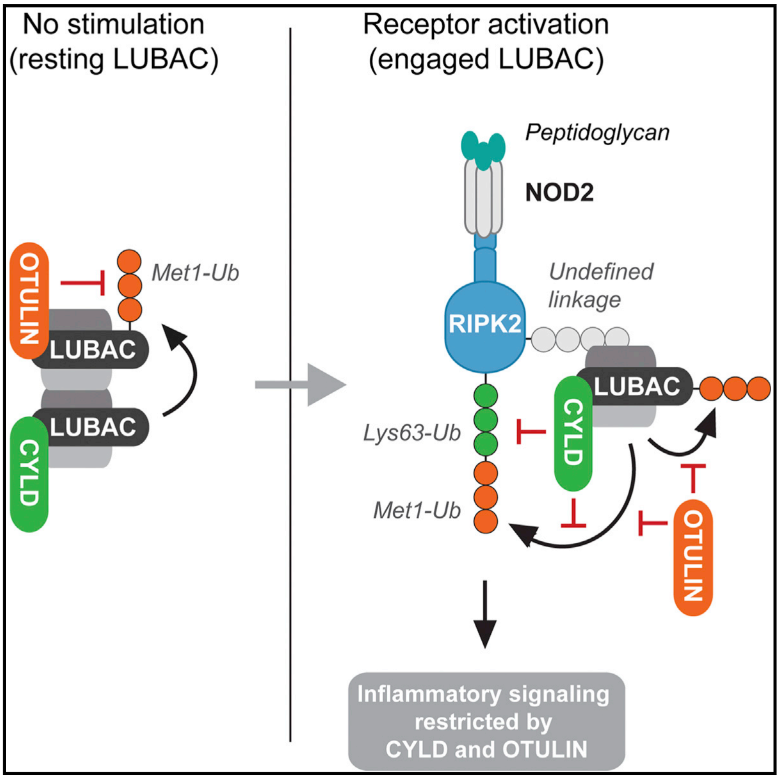

Highlights

- CYLD associates with LUBAC via HOIP and limits signaling by NOD2
- RIPK2 ubiquitination is regulated by CYLD and OTULIN
- CYLD trims Lys63 and Met1 linkages conjugated to RIPK2
- Productive NOD2 signaling requires Lys63 and Met1 linkages

Authors

Matous Hrdinka, Berthe Katrine Fiil, Mattia Zucca, ..., David Komander, Philipp J. Jost, Mads Gyrd-Hansen

Correspondence

mads.gyrd-hansen@ludwig.ox.ac.uk

In Brief

Hrdinka et al. show that productive signaling by the bacterial sensor NOD2 requires formation of Lys63- and Met1-linked ubiquitin. Both ubiquitin linkages are targeted by the deubiquitinase CYLD, which together with OTULIN regulates RIPK2 ubiquitination to restrict NOD2 signaling and cytokine production.

CrossMark

Hrdinka et al., 2016, Cell Reports *14*, 2846–2858
March 29, 2016 ©2016 The Authors
http://dx.doi.org/10.1016/j.celrep.2016.02.062

CellPress

OPEN ACCESS
CellPress

Cell Reports
Article

CYLD Limits Lys63- and Met1-Linked Ubiquitin at Receptor Complexes to Regulate Innate Immune Signaling

Matous Hrdinka,[1,4] Berthe Katrine Fiil,[1,4] Mattia Zucca,[1] Derek Leske,[1] Katrin Bagola,[1] Monica Yabal,[2] Paul R. Elliott,[3] Rune Busk Damgaard,[1,5] David Komander,[3] Philipp J. Jost,[2] and Mads Gyrd-Hansen[1,*]
[1]Ludwig Institute for Cancer Research, Nuffield Department of Clinical Medicine, University of Oxford, Old Road Campus Research Building, Oxford OX3 7DQ, UK
[2]III. Medizinische Klinik, Klinikum rechts der Isar, Technische Universität München, Munich 81675, Germany
[3]Medical Research Council Laboratory of Molecular Biology, Cambridge Biomedical Campus, Francis Crick Avenue, Cambridge CB2 0QH, UK
[4]Co-first author
[5]Present address: Medical Research Council Laboratory of Molecular Biology, Cambridge Biomedical Campus, Francis Crick Avenue, Cambridge CB2 0QH, UK
*Correspondence: mads.gyrd-hansen@ludwig.ox.ac.uk
http://dx.doi.org/10.1016/j.celrep.2016.02.062
This is an open access article under the CC BY license (http://creativecommons.org/licenses/by/4.0/).

SUMMARY

Innate immune signaling relies on the deposition of non-degradative polyubiquitin at receptor-signaling complexes, but how these ubiquitin modifications are regulated by deubiquitinases remains incompletely understood. Met1-linked ubiquitin (Met1-Ub) is assembled by the linear ubiquitin assembly complex (LUBAC), and this is counteracted by the Met1-Ub-specific deubiquitinase OTULIN, which binds to the catalytic LUBAC subunit HOIP. In this study, we report that HOIP also interacts with the deubiquitinase CYLD but that CYLD does not regulate ubiquitination of LUBAC components. Instead, CYLD limits extension of Lys63-Ub and Met1-Ub conjugated to RIPK2 to restrict signaling and cytokine production. Accordingly, Met1-Ub and Lys63-Ub were individually required for productive NOD2 signaling. Our study thus suggests that LUBAC, through its associated deubiquitinases, coordinates the deposition of not only Met1-Ub but also Lys63-Ub to ensure an appropriate response to innate immune receptor activation.

INTRODUCTION

Ubiquitin (Ub) chains linked via the N-terminal methionine (Met1) of Ub (Met1-Ub, also termed linear Ub) and lysine 63 (Lys63-Ub) facilitate innate immune signaling initiated by pattern recognition receptors (PRRs) such as toll-like receptors (TLRs) and nucleotide-oligomerization domain (NOD)-like receptors and cytokine receptors such as tumor necrosis factor (TNF) receptor 1 (TNFR1) (Fiil and Gyrd-Hansen, 2014; Jiang and Chen, 2012). The linear Ub chain assembly complex (LUBAC), composed of HOIL-1, HOIP, and SHARPIN, is the only known Ub ligase to generate Met1-Ub (Gerlach et al., 2011; Ikeda et al., 2011; Kirisako et al., 2006; Tokunaga et al., 2011). LUBAC activity is counterbalanced by the Met1-specific deubiquitinase (DUB) OTULIN (Fiil et al., 2013; Keusekotten et al., 2013; Rivkin et al., 2013), which binds to the catalytic subunit HOIP via interactions between the HOIP peptide:N-glycanase/UBA- or UBX-containing proteins (PUB) domain and a PUB-interacting motif (PIM) in OTULIN (Elliott et al., 2014; Schaeffer et al., 2014). The importance of Met1-Ub in immune signaling is underscored by identification of mutations within the LUBAC-encoding genes in human patients with immunological disease (Boisson et al., 2012, 2015). Lys63-Ub can be generated by Ub ligases that interact with the dimeric E2 complex Ubc13/Uev1a, which exclusively conjugates this linkage (Deng et al., 2000). Lys63-Ub is particularly important in MyD88-dependent immune-signaling pathways activated by TLRs and interleukin-1 receptors (IL-1R) whereas the role of Lys63-Ub in the NOD-containing protein 2 (NOD2) and TNFR1 pathways is not fully understood (Fiil and Gyrd-Hansen, 2014; Xu et al., 2009).

NOD2 is an intracellular bacteria-sensing PRR that recognizes MDP (muramyl dipeptide) constituents of bacterial peptidoglycan and plays a critical role in gastro-intestinal immunity (Philpott et al., 2014). Upon stimulation, NOD2 binds receptor-interacting protein kinase 2 (RIPK2, also known as RIP2 or RICK), leading to recruitment of several Ub ligases including the inhibitor of apoptosis (IAP) proteins, cIAP1, cIAP2, and XIAP (Bertrand et al., 2009; Damgaard et al., 2012). XIAP is indispensable for NOD2 pathway functionality, where it ubiquitinates RIPK2 to facilitate recruitment of LUBAC (Bauler et al., 2008; Damgaard et al., 2012). In turn, LUBAC assembles Met1-Ub on RIPK2 to enable downstream signal transduction (Fiil et al., 2013). Additionally, TRAF2, ITCH, cIAP1/2, TRAF6, and PELI3 are reported to contribute to the assembly of Lys63-Ub on RIPK2, but their individual contribution to this process and to NOD2 signaling is not fully resolved (Bertrand et al., 2009; Hasegawa et al., 2008; Tao

et al., 2009; Watanabe et al., 2014; Yang et al., 2013). A central regulatory point for productive innate immune signaling and transcription of nuclear factor-κB (NF-κB) target genes is the activation of the IKK (IκB kinase) complex. IKK activation is dependent on phosphorylation by the TAB/TAK1 complex that interacts with Lys63-Ub and on the conjugation of Met1-Ub by LUBAC, which is bound by the IKK subunit NEMO (also known as IKKγ; Fiil and Gyrd-Hansen, 2014; Jiang and Chen, 2012).

For appropriate and beneficial innate immune signaling, the assembly of Ub chains at receptor complexes must be carefully counterbalanced by DUBs. The linkage-selective DUBs OTULIN, CYLD, and A20 regulate various aspects of pro-inflammatory signaling (Fiil and Gyrd-Hansen, 2014; Harhaj and Dixit, 2012). The A20 gene (*TNFAIP3*) is a transcriptional target of NF-κB and A20 functions as a part of the negative feedback mechanism to terminate signaling (Harhaj and Dixit, 2012; Lee et al., 2000). Contrary to this, OTULIN functions to restrict the accumulation of Met1-Ub at basal conditions and early during signaling, and *OTULIN* expression is not induced by stimulation of NF-κB activity (Fiil et al., 2013; Keusekotten et al., 2013; Rivkin et al., 2013).

CYLD is a bona fide tumor suppressor and negatively regulates pro-inflammatory signaling (Bignell et al., 2000; Harhaj and Dixit, 2012). CYLD belongs to the USP (Ub-specific protease) family of DUBs (Brummelkamp et al., 2003; Kovalenko et al., 2003; Trompouki et al., 2003) and in vitro cleaves Lys63-Ub and Met1-Ub with similar efficiency while displaying less activity toward Lys11-Ub and Lys48-Ub (Komander et al., 2009; Ritorto et al., 2014; Sato et al., 2015). Unexpectedly, CYLD was recently reported to interact with HOIP, the catalytic subunit of LUBAC, and to inhibit LUBAC-dependent activation of NF-κB (Takiuchi et al., 2014).

Here, we show that, although CYLD is associated with LUBAC through HOIP binding, CYLD does not regulate ubiquitination of LUBAC components. Instead, CYLD counteracts Lys63-Ub and Met1-Ub conjugated to the LUBAC substrate RIPK2 to restrict signaling and cytokine production. Our results suggest that LUBAC not only is a Met1-specific E3 but also, through its associated DUBs, coordinates Met1- and Lys63-linked Ub chain assembly at signaling complexes.

RESULTS

CYLD Antagonizes LUBAC Function but Does Not Affect HOIP Ubiquitination

LUBAC function is restricted by OTULIN through its docking to the PUB domain of HOIP (Elliott et al., 2014; Schaeffer et al., 2014). Unexpectedly, CYLD also interacts with LUBAC via the HOIP PUB domain and mutations, such as N102D, that interfere with OTULIN binding also interfere with CYLD binding (Takiuchi et al., 2014; Figures 1A–1C). Moreover, CYLD can, akin to OTULIN, suppress LUBAC-induced NF-κB activation through its DUB activity (Takiuchi et al., 2014; Figures 1D and S1A). This together with the fact that CYLD cleaves Lys63-Ub and Met1-Ub in vitro (Komander et al., 2009; Sato et al., 2015; Figure S1B) implies that CYLD and OTULIN might function in a similar manner to control Met1-Ub conjugation by LUBAC.

To investigate this, we initially tested whether CYLD, like OTULIN, regulates HOIP ubiquitination. Surprisingly, depletion of CYLD in U2OS/NOD2 cells (a cell line expressing doxycycline [DOX]-inducible HA-NOD2 that responds to the NOD2 ligand L18-MDP without addition of DOX due to slight leakiness of the promoter; Fiil et al., 2013) had no effect on HOIP ubiquitination under basal conditions or after receptor stimulation (Figures 1E and S1C). In contrast, OTULIN depletion led to extensive accumulation of Met1-Ub on HOIP (Figures 1E and S1C). Affinity purification of Ub conjugates via linkage-specific Ub binders (SUBs) selective for Met1-Ub (M1-SUB; Fiil et al., 2013) or Lys63-Ub (K63-SUB; Sims et al., 2012; Thorslund et al., 2015) showed that Met1-Ub, but not Lys63-Ub, accumulated on LUBAC (Figure 1E). Analysis of THP1 human monocytic cells or HCT-116 human colon carcinoma cells, which both express NOD2 endogenously, confirmed that CYLD was not involved in controlling Met1-Ub accumulation on HOIP whereas OTULIN was indispensable (Figures 1F, 1G, S1D, and S1E). Accordingly, ectopic expression of inactive OTULIN (C129A), but not inactive CYLD (C601A), caused extensive accumulation of Met1-Ub on HOIP (Figures 1H and S1F). This prompted us to explore the role of CYLD in regulating the NOD2 pathway, which relies on LUBAC.

CYLD Activity Controls NOD2 Signaling

CYLD has been reported to inhibit RIPK2-induced signaling when overexpressed (Abbott et al., 2004), but the function of endogenous CYLD in NOD2 signaling remains unexplored and its role as a DUB is unknown. Depletion of CYLD showed that CYLD limits productive signaling after NOD2 stimulation as determined by accumulation of transcripts from the NF-κB-responsive genes *TNF* and *CXCL8* and production of IL-8 (encoded by *CXCL8*; Figures 2A and 2B). The requirement for CYLD in restricting NOD2 responses was further validated in CYLD-deficient bone-marrow-derived dendritic cells (BMDCs), which produced markedly higher levels of pro-inflammatory cytokines than their wild-type counterparts upon NOD2 activation (Figure 2C).

OTULIN depletion also led to increased IL-8 production, but the effect was less pronounced as compared to CYLD depletion (Figures 2D and S2A). Because both DUBs cleave Met1-Ub, we tested whether depletion of both DUBs would further deregulate NOD2 signaling. However, we found no additive or synergistic effect on IL-8 production or on MAP kinase and IKK signaling when both enzymes were depleted as compared to their individual depletion (Figures 2D, S2A, and S2B). Interestingly, the depletion of CYLD did not affect IL-8 production after stimulation with TNF (Figure 2B), which might reflect a differential dependency of NOD2- and TNFR1-signaling pathways on LUBAC function (Damgaard et al., 2012; Gerlach et al., 2011). To test this, the transcriptional response to NOD2 and TNFR1 stimulation was tested in HOIP-deficient HCT-116 (HOIP-KO) cells (Figure 2E). Transcriptional activation of *CXCL8* was ablated in HOIP-KO cells after NOD2 stimulation but was only reduced after TNF stimulation (Figure 2F). In fact, *CXCL8* was induced more than 25-fold in HOIP-KO cells in response to TNF. Also, measurement of NF-κB activity by a luciferase-based reporter showed that HOIP is required for NOD2-dependent NF-κB activation whereas TNF-induced NF-κB activation in HOIP KO cells is only partially decreased (Figure S2C). Reconstitution of the HOIP-KO cells with ectopic HOIP restored NF-κB activation in

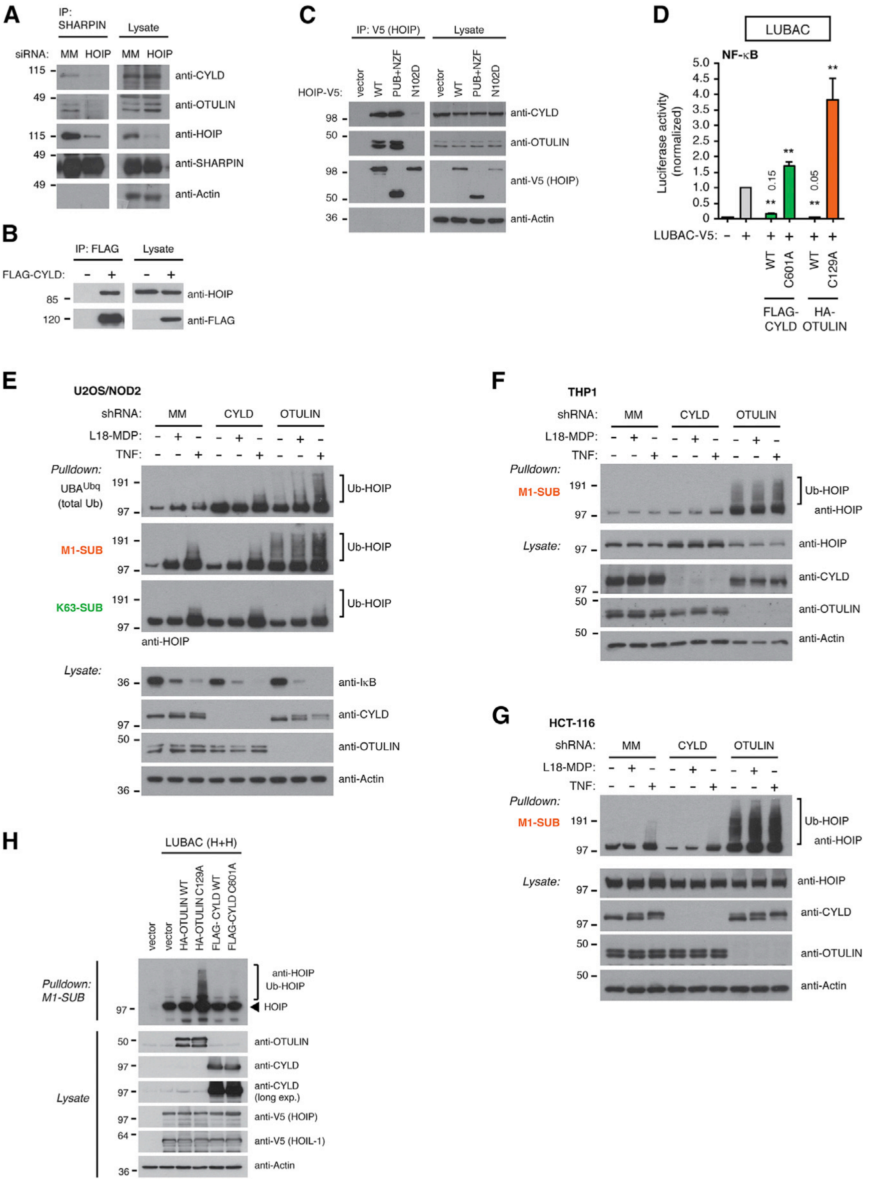

Figure 1. CYLD Antagonizes LUBAC Function but Does Not Affect HOIP Ubiquitination

(A) Immunoprecipitation of endogenous SHARPIN from control (MM) and HOIP-depleted U2OS/NOD2 cells. Lysates and immunoprecipitated material were examined by immunoblotting.

(B) Immunoprecipitation of exogenous FLAG-tagged CYLD. Lysates and immunoprecipitated material were examined for copurification of HOIP.

(C) Immunoprecipitation of exogenous V5-tagged $HOIP^{WT}$, $HOIP^{PUB+NZF}$, and $HOIP^{N102D}$ expressed in HEK293FT cells. Immunoprecipitated material was examined for copurification of OTULIN and CYLD.

(D) NF-κB activity in HEK293FT cell lysates transfected with dual luciferase reporters, co-expressed with vector LUBAC (HOIL-1/HOIP), OTULIN, or CYLD variants as indicated. Luciferase activity is shown relative to the activity in LUBAC-transfected cells.

(E) Purification of endogenous Ub conjugates using UBA^{Ubq}, M1-SUB, or K63-SUB in U2OS/NOD2 cell lysates stably depleted for CYLD or OTULIN and treated with L18-MDP (200 ng/ml; 1 hr) or TNF (10 ng/ml; 10 min). Purified material and lysates were examined by immunoblotting.

(F and G) Purification of endogenous Ub conjugates in THP1 cells (F) or HCT-116 cells (G) stably depleted for CYLD or OTULIN and treated with L18-MDP (200 ng/ml; 1 hr) or TNF (10 ng/ml; 10 min). Purified material and lysates were examined by immunoblotting.

(H) Purification of endogenous Ub conjugates in HEK293FT cell lysates transfected with OTULIN or CYLD variants co-expressed with LUBAC as indicated. Purified material and lysates were examined by immunoblotting.

Data in (D) represent the mean ± SEM of at least three independent experiments, each performed in duplicate. $^{**}p < 0.01$. See also Figure S1.

response to L18-MDP and TNF, showing that the signaling defect of the HOIP-KO cells was caused by the absence of HOIP (Figure S2C).

Next, we investigated the role of the DUB activity of CYLD in regulating NOD2 signaling. For this, the effect of wild-type CYLD ($CYLD^{WT}$) and catalytic inactive CYLD ($CYLD^{C601A}$) on nuclear translocation of the NF-κB subunit RelA and the production of IL-8 was determined. This showed that the ability of CYLD to antagonize productive NOD2 signaling relied on its catalytic activity (Figures 3A–3C, S3A, and S3B). Also, activation of NF-κB by ectopic XIAP, an essential Ub ligase for NOD2 signaling, was blocked by overexpression of $CYLD^{WT}$, but not $CYLD^{C601A}$, which increased NF-κB activity (Figures 3D and S3C). XIAP-induced NF-κB activity relies not exclusively on Met1-Ub (Damgaard et al., 2012) but was also dependent on Ubc13-mediated formation of Lys63-Ub (Figures 3E and S3D), which might explain why OTULIN only partially inhibited XIAP-induced NF-κB activity (Figure 3D). CYLD and OTULIN were not inhibiting NF-κB activity per se because the DUBs did not appreciably inhibit NF-κB activity induced by an engineered non-cleavable Met1-Ub4 protein targeted to inactive XIAP (Fiil et al., 2013; Figures S3E and S3F).

CYLD Limits Extension of Ub Chains on RIPK Proteins

Because CYLD functioned as a DUB to limit NOD2-dependent signaling but did not regulate LUBAC ubiquitination, we asked whether depletion of CYLD would affect LUBAC substrate ubiquitination in response to receptor stimulation. Indeed, purification of Ub conjugates from control and CYLD-depleted cells stimulated with L18-MDP revealed that CYLD-depleted cells accumulated Ub-RIPK2 species containing Lys63 and Met1 linkages with a higher apparent molecular weight (MW) than observed in control cells, particularly at early time points (Figures 4A and S4A). The effect of CYLD depletion on Ub-RIPK2 was strikingly different from the effect of OTULIN depletion, which

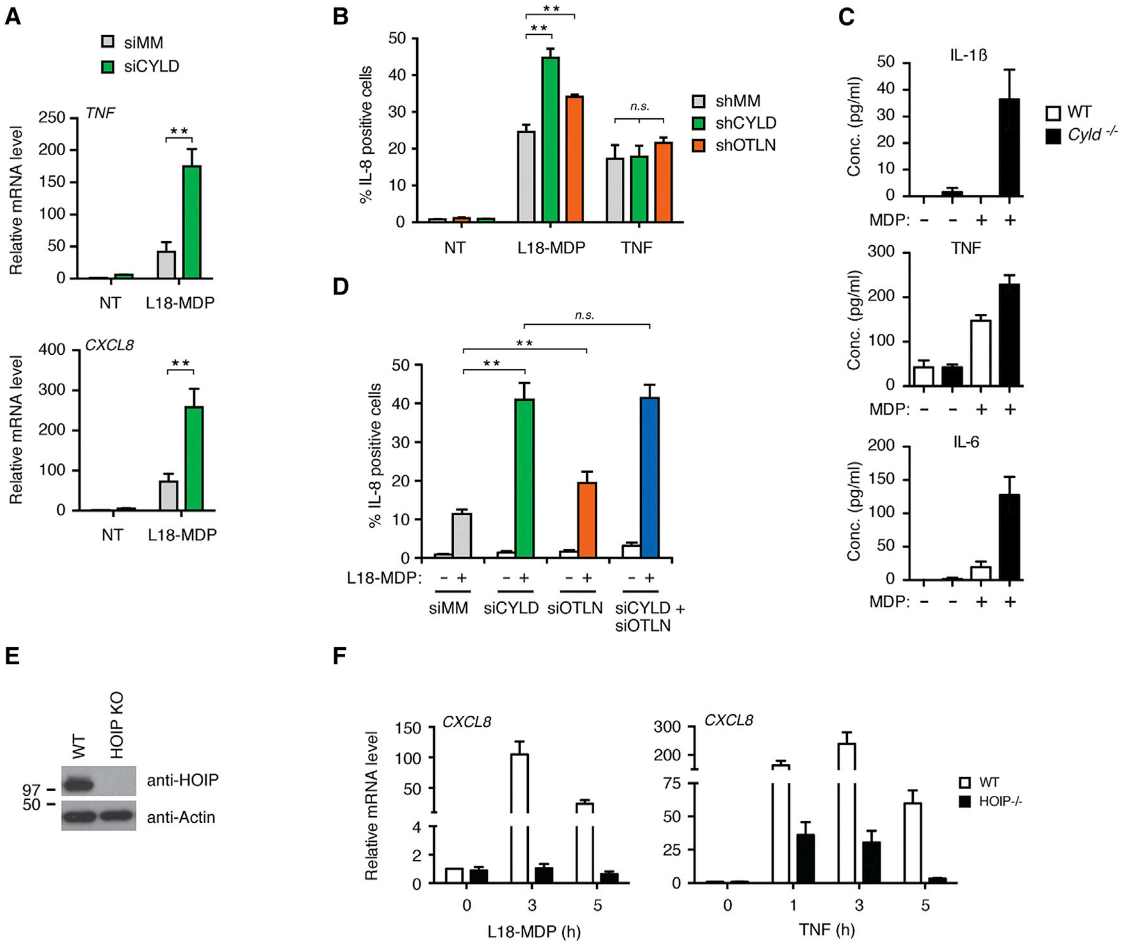

Figure 2. CYLD Restricts NOD2 Signaling and Cytokine Production

(A) Relative levels of *TNF* and *CXCL8* transcripts from U2OS/NOD2 control (siMM) and CYLD-depleted (siCYLD) cells treated with L18-MDP (200 ng/ml; 3 hr) normalized to untreated siMM.

(B) Intracellular flow cytometry analysis of IL-8 in shRNA control (shMM) U2OS/NOD2 cells or cells stably depleted for OTULIN (shOTLN) or CYLD (shCYLD) in response to L18-MDP (200 ng/ml; 4 hr) or TNF (10 ng/ml; 4 hr).

(C) WT and $Cyld^{-/-}$ BMDCs were stimulated with MDP (10 μg/ml; 24 hr), and secreted cytokines were measured in culture supernatants.

(D) Intracellular flow cytometry analysis of IL-8 in U2OS/NOD2 cells depleted for OTULIN (siOTLN) and CYLD (siCYLD) or control (siMM) using siRNA oligos in response to L18-MDP (200 ng/ml; 4 hr).

(E) Immunoblot of HOIP levels in control HCT-116 cells and in CRISPR/Cas9 HOIP KO cells.

(F) Relative levels of *CXCL8* transcripts from HCT-116 WT and HOIP KO cells treated with L18-MDP (200 ng/ml) and TNF (10 ng/ml) for the indicated times and normalized to untreated control cells. Data are shown on a two-segmented y axis.

Data in (A), (B), (D), and (F) represent the mean ± SEM of at least three independent experiments, each performed in duplicate. **$p < 0.01$; *n.s.* not significant. See also Figure S2.

promoted the accumulation of Ub-RIPK2 species but did not affect the MW of Ub-RIPK2 as compared with control cells (Fiil et al., 2013; Figure 4B).

CYLD is reported to be a transcriptional target of NF-κB in response to TNF and IL-1β treatment and to contribute to termination of signaling (Jono et al., 2004). However, depletion of CYLD did not stabilize RIPK2 ubiquitination at late time points after NOD2 stimulation (Figure 4A). Also, NOD2 stimulation did not increase *CYLD* (or *OTULIN*) mRNA or protein levels whereas *TNFAIP3* (A20) mRNA and protein levels rapidly increased by the treatment (Figures 4C and 4D). This suggested that CYLD regulates ubiquitination at the initiation of signaling. In line with this, TNF treatment of CYLD-depleted cells led to accumulation of Ub-RIPK1 species with a higher apparent MW than observed in non-depleted cells within 10 min (Figure 4E), which coincided with the recruitment of CYLD to the TNFR-SC (Figure 4F). CYLD also co-purified with HA-NOD2 induced by DOX in U2OS/NOD2 cells, and under these conditions, CYLD levels were unaffected by the treatment (Figure 4G).

The ubiquitination of RIPK2 and RIPK1 after NOD2 and TNFR1 stimulation, respectively, is facilitated by several E3 Ub ligases, including XIAP and cIAPs. The continuous assembly of Ub chains within receptor-signaling complexes might therefore mask the regulation of ubiquitination by DUBs. To better assess

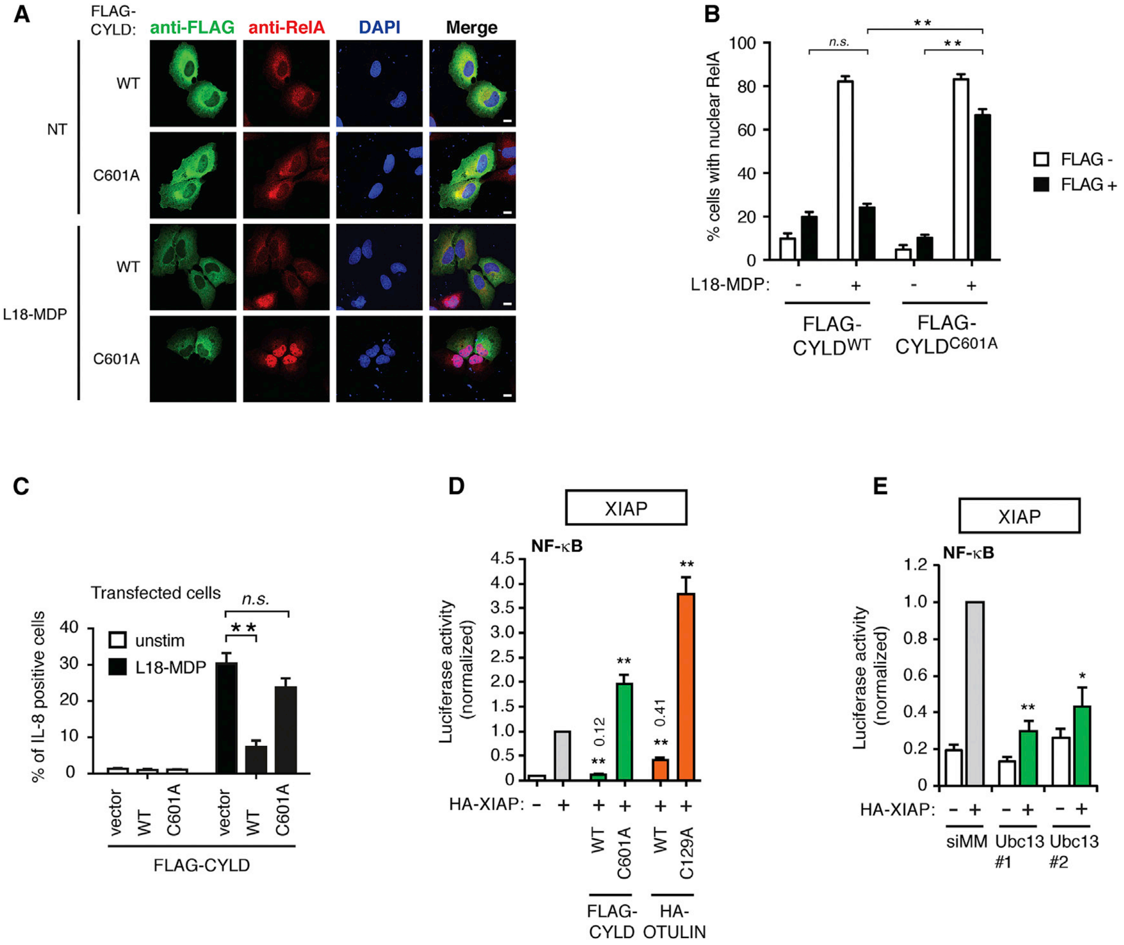

Figure 3. CYLD Catalytic Activity Inhibits the NOD2 Pathway Upstream of Nuclear Translocation of NF-κB

(A) Immunofluorescence analysis of nuclear translocation of the NF-κB subunit RelA/p65 (red) in response to L18-MDP (1 μg/ml; 1 hr) in U2OS/NOD2 cells transfected with FLAG-CYLD variants and stained with anti-FLAG (green; scale bar, 10 μm).

(B) Quantification of cells with nuclear RelA treated as in (A).

(C) Intracellular flow cytometry analysis of IL-8 in U2OS/NOD2 cells transfected with FLAG-CYLD variants in response to L18-MDP (200 ng/ml; 4 hr). Cells were cotransfected with a GFP vector (ratio 1:10) as a marker of transfection.

(D) NF-κB activity in HEK293FT cell lysates transfected with dual luciferase reporters, XIAP, CYLD, and OTULIN as indicated. Values are expressed relative to XIAP transfection.

(E) NF-κB activity in HEK293T cell lysates transfected with luciferase reporters, vector, or XIAP and depleted for Ubc13 using two different siRNAs. Values are expressed relative to XIAP transfection.

Data in (B)–(E) represent the mean ± SEM of at least three independent experiments, each performed in duplicate. **$p < 0.01$; *$p < 0.05$. See also Figure S3.

the regulation of Ub chains by CYLD, we therefore treated cells with a Smac-mimetic compound (compound A [CpA]) to inhibit IAP function prior to receptor stimulation. When used at high concentrations (1 μM), CpA blocks RIPK2 ubiquitination in response to L18-MDP (Figures 5A, lanes 1–4, and S5A) because the compound antagonizes the interaction of XIAP with RIPK2 in addition to inducing degradation of cIAPs (Damgaard et al., 2013). Remarkably, depletion of CYLD under these conditions restored RIPK2 ubiquitination to comparable levels as in NOD2-stimulated cells not treated with CpA (Figure 5A, compare lane 8 with lanes 3 and 7). Depletion of OTULIN also led to accumulation of Ub-RIPK2 albeit to a lesser extent than when CYLD was depleted (Figure 5A, compare lane 12 with lanes 3 and 11). CpA also impaired the ubiquitination of RIPK1 in response to TNF, and depletion of CYLD partially restored RIPK1 ubiquitination (Figures 5B, compare lanes 4 and 8, and S5B). This suggests that CYLD and OTULIN, especially at the NOD2 receptor complex, are highly active in regulating Ub chain stability.

CpA prevented the degradation of IκBα by L18-MDP in non-depleted cells but in CYLD-depleted cells IκBα levels were reduced after stimulation, suggesting that CYLD depletion restored productive signaling (Figure 5A, compare lanes 4 and 8). However, CpA inhibited IL-8 production in CYLD-depleted cells to a similar extent as in in control or OTULIN-depleted cells (Figure 5C). In contrast, CpA had no effect on IL-8 induced by phorbol myristate acetate (PMA) and ionomycin, indicating that the compound was not interfering with IL-8 production non-specifically (Figure 5D).

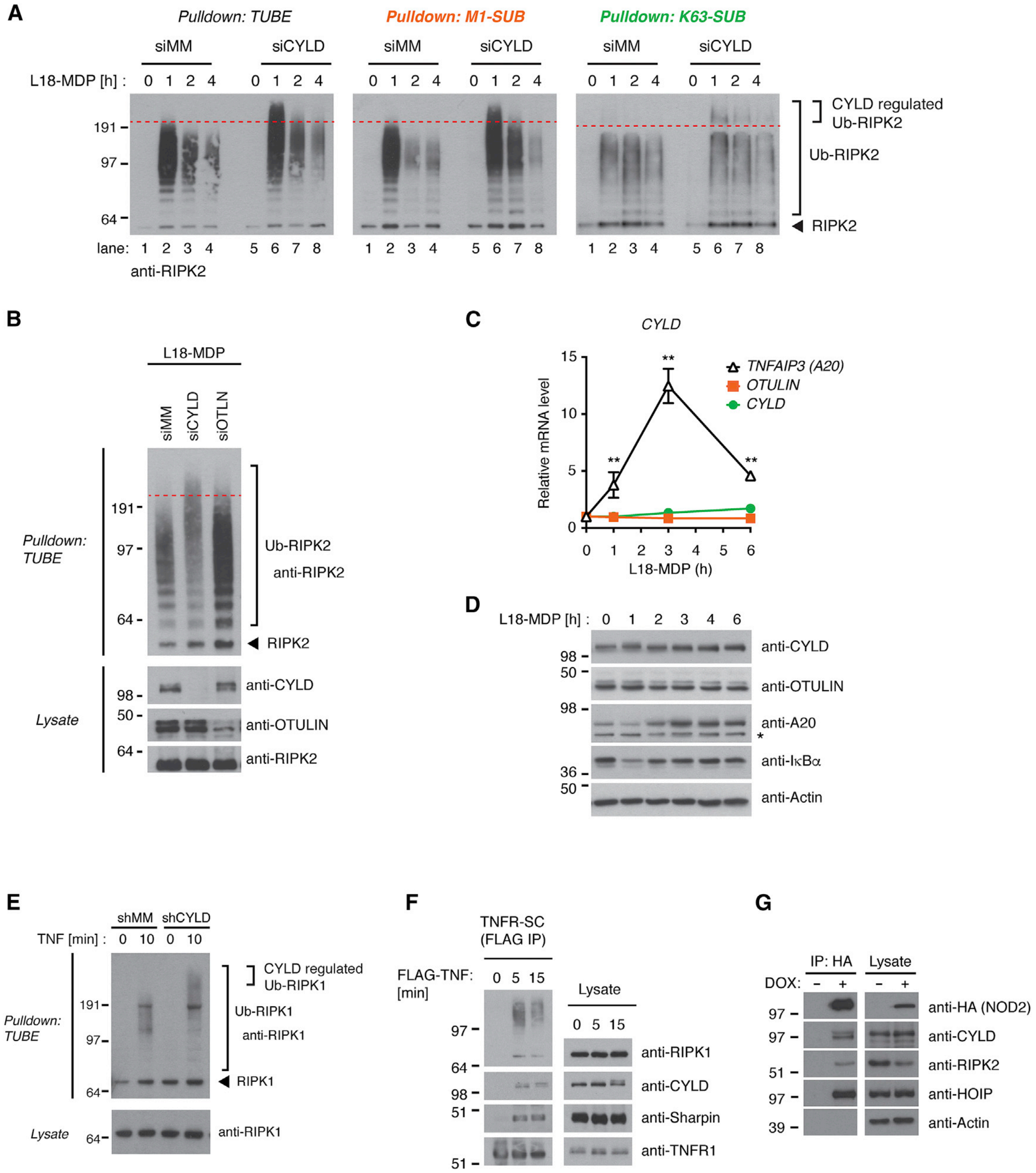

Figure 4. CYLD Limits Extension of Ub Chains on RIPK Proteins

(A) Purification of Ub conjugates from U2OS/NOD2 cells at indicated time points after treatment with L18-MDP (200 ng/ml). Purified material was examined for ubiquitinated RIPK2 by immunoblotting.

(B) Purification of Ub conjugates from control or OTULIN- or CYLD-depleted U2OS/NOD2 cells treated with L18-MDP (200 ng/ml; 1 hr). Purified material and lysate were examined by immunoblotting.

(C and D) Relative mRNA (C) and protein (D) levels of *CYLD*, *OTULIN*, and *TNFAIP3* (*A20*) upon L18-MDP stimulation (200 ng/ml) of U2OS/NOD2 cells at the time points indicated. Asterisk denotes unspecific band detected by the antibody.

(E) Purification of Ub conjugates from U2OS/NOD2 cells treated with TNF (10 ng/ml; 10 min). Purified material and lysate were examined for RIPK1 by immunoblotting.

(F) Purification of TNFR-SC from U2OS/NOD2 cells stimulated with FLAG-TNF using anti-FLAG agarose and analyzed by immunoblotting.

(G) Immunoprecipitation of HA-NOD2 from U2OS/NOD2 cells. HA-NOD2 expression was induced with DOX (2 μg/ml) for 24 hr. Immunoprecipitates were examined for co-purification of CYLD and other members of the NOD2 receptor complex.

Data in (C) represent the mean ± SEM of at least three independent experiments, each performed in duplicate. **$p < 0.01$. See also Figure S4.

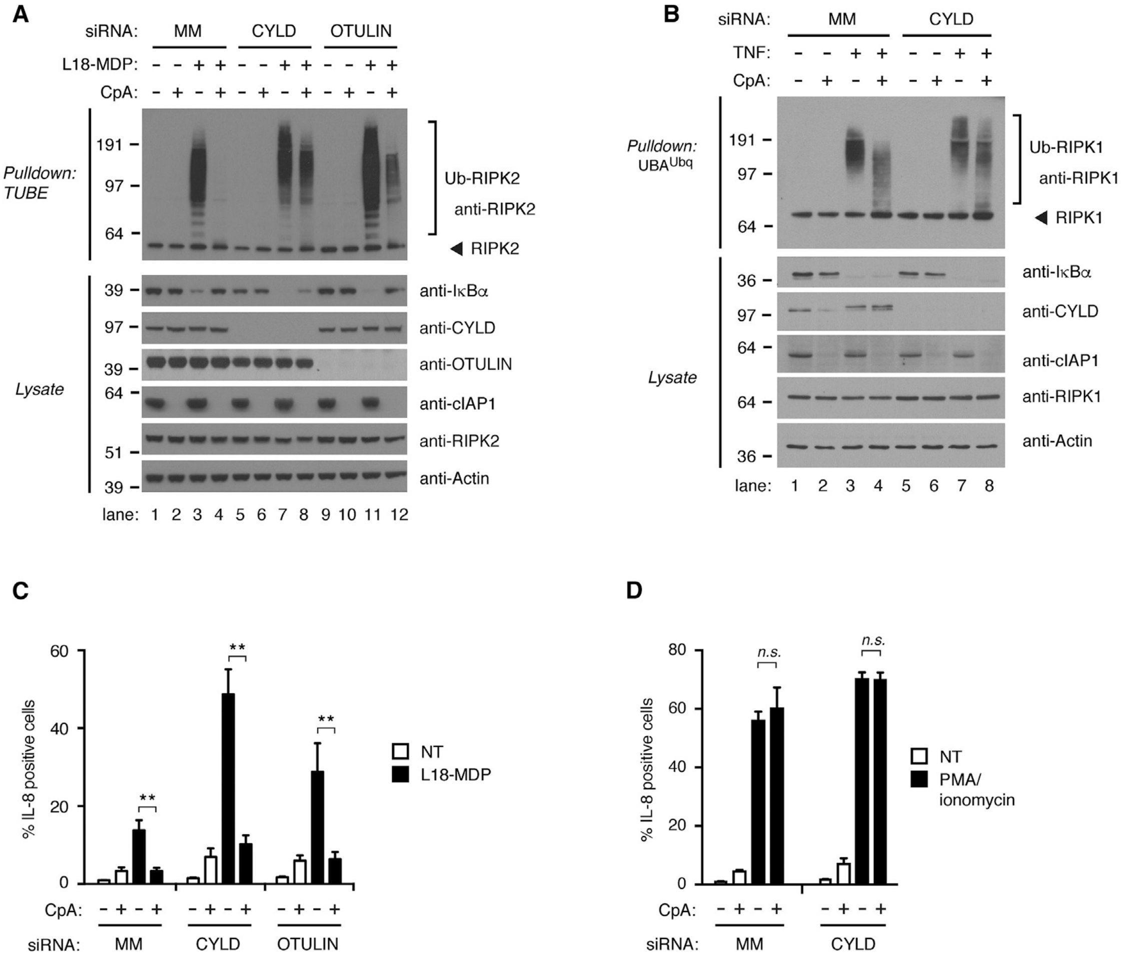

Figure 5. Inhibition of IAPs Reveals Extensive Regulation of RIPK2 Ubiquitination by CYLD and OTULIN

(A and B) Purification of endogenous Ub conjugates from U2OS/NOD2 cells depleted for CYLD or OTULIN by siRNA. Cells were pre-treated with DMSO (control) or with 1 μM compound A (CpA) for 30 min before stimulation with (A) L18-MDP (200 ng/ml; 1 hr) or (B) TNF (10 ng/ml; 10 min).

(C and D) Intracellular flow cytometry analysis of IL-8 in control (MM) U2OS/NOD2 cells or cells depleted for CYLD or OTULIN by siRNA pre-treated with 1 μM CpA for 30 min before stimulation with (C) L18-MDP (200 ng/ml; 4 hr) or (D) combination of phorbol myristate acetate (PMA) (50 ng/ml) and ionomycin (500 μM) for 4 hr. Data in (C) and (D) represent the mean ± SEM of at least three independent experiments, each performed in duplicate. **p < 0.01.

See also Figure S5.

CYLD Trims Lys63- and Met1-Ub on RIPK2

To address which Ub-linkage(s) CYLD regulates in the context of NOD2 pathway activation, we subjected Ub conjugates isolated with glutathione S-transferase (GST)-M1-SUB (Figure 6A) or GST-UBA^{Ubq} (Figure 6B) to Ub chains restriction (UbiCRest) analysis (Hospenthal et al., 2015). Incubation with OTULIN^{WT}, but not inactive OTULIN^{C129A}, resulted in a gel shift of Ub-RIPK2, but the digested Ub-RIPK2 from CYLD-depleted cells still migrated significantly slower than Ub-RIPK2 from control cells (Figures 6A, compare lanes 1 and 3 with lanes 7 and 9, and 6B, compare lanes 2 and 5 with lanes 9 and 12). Additional Met1 linkages could thus not alone account for the slower migrating Ub-RIPK2 species in CYLD-depleted cells. Incubation with the Lys63-selective DUBs OTUD1 or AMSH* (Michel et al., 2015) also resulted in a similar gel shift of Ub-RIPK2 isolated from control and CYLD-depleted cells (Figure 6B, compare lanes 2, 3, and 6 with lanes 9, 10, and 13). However, incubation with OTULIN plus OTUD1 or OTULIN plus AMSH* resulted in similar migration patterns of Ub-RIPK2 isolated from control and CYLD-depleted cells, indicating that CYLD restricts the deposition of both Lys63 and Met1 linkages on RIPK2 (Figure 6B, compare lanes 4 and 7 with lanes 11 and 14). The extent of Ub chain digestion and linkage specificity in the DUB reactions was determined by linkage-specific antibodies and by spiking into the reaction recombinant Met1-linked Ub4 with an N-terminal GST moiety. This indicated that OTUD1 and OTULIN cleaved the intended linkage largely to completion without detectable cross-reactivity with Met1- or Lys63-Ub, respectively (Figures S6A and S6B). Curiously, a substantial fraction of the high-MW signal detected by the Met1-Ub antibody disappeared in the OTUD1-treated samples even though OTUD1 did not digest GST-Met1-Ub4. This could reflect that Met1 linkages in general are conjugated to existing Lys63-Ub although the remaining signal in the OTULIN-treated samples suggests that the

OPEN ACCESS
CellPress

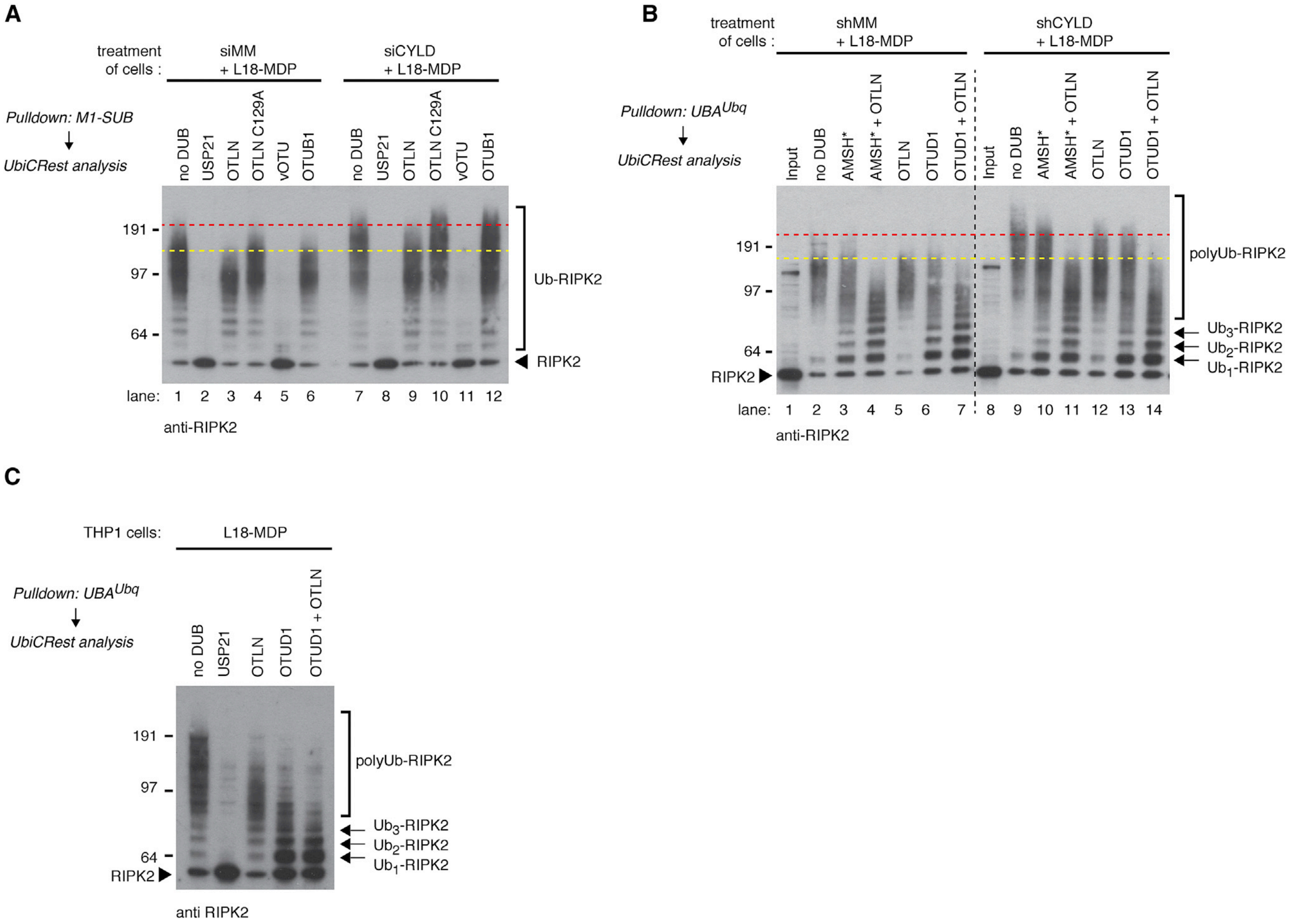

Figure 6. CYLD Trims Lys63- and Met1-Ub on RIPK2

(A) UbiCRest analysis of ubiquitinated RIPK2 isolated with M1-SUB from L18-MDP-treated (200 ng/ml; 1 hr) control (siMM) or CYLD-depleted (siCYLD) U2OS/NOD2 cells. Purified Ub conjugates were incubated with the indicated DUBs for 1 hr, and samples were examined by immunoblotting.

(B) As in (A) except ubiquitinated RIPK2 was isolated with GST-UBA^{Ubq} followed by incubation with the indicated DUBs for 15 min and cells were stably depleted for CYLD (shCYLD) or control (shMM). Black dashed line separates two scans of the same membrane but with slightly different exposure.

(C) As in (B) except that Ub conjugates were purified from THP-1 cells and incubated for 1 hr.

See also Figure S6.

Met1-Ub antibody also can react with Ub chains other than Met1-Ub (Figure S6A).

Notably, incubation of the purified Ub conjugates with AMSH* or OTUD1, but not with OTULIN, generated significant amounts of monoUb-RIPK2 and oligoUb-RIPK2, suggesting that Lys63 linkages are generated proximal to RIPK2 whereas Met1 linkages are only conjugated to polyubiquitinated RIPK2 (Figures 6B, 6C, and S6A–S6C). The viral DUB vOTU disassembles all Ub linkages except Met1 (Hospenthal et al., 2015) and served as a positive control along with USP21 in the UbiCREST analysis, where they removed virtually all Ub moieties from RIPK2 (Figures 6A and 6C).

Lys63-Ub and Met1-Ub Are Individually Indispensable for NOD2 Signaling

Lys63-Ub had been suggested to contribute to NOD2 signaling, and our investigation of CYLD's function supported this notion. However, the direct evidence for the requirement of the Lys63 linkage was still lacking. To address this, we expressed GFP-coupled variants of the K63-SUB and M1-SUB (Figure 7A) in order to interfere with the function of Lys63-Ub and Met1-Ub, respectively, as was previously reported (Fiil et al., 2013; Sims et al., 2012). Indeed, expression of GFP-K63-SUB inhibited the nuclear localization of RelA and production of IL-8 after NOD2 stimulation to a similar extent as GFP-M1-SUB despite being expressed at lower levels (Figures 7B–7D, S7A, and S7B). Gating of cells based on GFP expression revealed that low levels of GFP-K63-SUB were sufficient to block IL-8 production whereas GFP-M1-SUB inhibited IL-8 production only when highly expressed (Figure 7E). As expected, GFP-negative cells (not successfully transfected) responded similarly to NOD2 stimulation in all conditions (Figures 7C and 7E).

A substantial fraction of GFP-K63-SUB appeared to localize to the nucleus whereas GFP-M1-SUB was more evenly distributed

between cytoplasm and nucleus (Figure 7B). We therefore generated variants with a nuclear localization signal (NLS) and transfected cells with nuclear-localized variants. This showed that the GFP-SUBs exclusively inhibited signaling in the cytoplasm because neither NLS-GFP-K63-SUB nor NLS-GFP-M1-SUB inhibited IL-8 production (Figures 7F and S7A).

Consistent with an important function of Lys63-Ub for NOD2 signaling, depletion of Ubc13 inhibited IL-8 production to a similar extent as the depletion of HOIP (Figure 7G). Similar effects were obtained in cells where either CYLD or OTULIN was stably silenced, albeit the inhibitory effect of Ubc13 or HOIP depletion on IL-8 production was slightly less effective and depletion of both proteins was needed to completely prevent IL-8 production (Figure 7G). Altogether, our data reveal that the NOD2 pathway is exquisitely dependent on Lys63-Ub and Met1-Ub and suggest that the regulation of these linkages is coordinated by LUBAC through its associated DUBs CYLD and OTULIN.

DISCUSSION

In this study, we revealed that CYLD restricts deposition of Lys63-Ub and Met1-Ub on the LUBAC substrate RIPK2 to limit NOD2-dependent inflammatory signaling. We showed that CYLD, like OTULIN, interacts with LUBAC, suggesting that the Ub-regulating capacity of LUBAC complexes extends beyond Met1-Ub to also include Lys63-Ub.

There are several examples of Ub ligase-DUB pairs where a DUB either regulates substrate ubiquitination or auto-ubiquitination of the Ub ligase, notably the Mdm2-USP7 complex that regulates p53 stability and the DUB A20, which also harbors Ub ligase activity (Tang et al., 2006; Wertz et al., 2004). However, we are not aware of other examples than LUBAC-OTULIN-CYLD, where an Ub ligase associates with two separate DUB activities. It is possible that LUBAC exists as different complexes containing either CYLD or OTULIN because they both require residues within the PIM-binding pocket (e.g., N102) for interaction with HOIP. However, the LUBAC complex elutes as an ~600-kDa complex and may thus contain two or more HOIP molecules, allowing for binding of both DUBs within the same complex (Kirisako et al., 2006). Supporting the latter scenario, HOIP oligomerizes via the N-terminal part of the protein (Elliott et al., 2014), and CYLD has been shown to co-immunoprecipitate OTULIN in a LUBAC-dependent manner and vice versa (Takiuchi et al., 2014). Moreover, we found that the removal of Met1-Ub conjugated to HOIP was independent of CYLD but was entirely dependent on OTULIN's catalytic activity and its binding to LUBAC (Elliott et al., 2014), arguing for the existence of a ternary LUBAC-CYLD-OTULIN complex.

The molecular basis preventing CYLD from processing Met1-Ub on HOIP is not clear, but it could be via inaccessibility of CYLD, but not OTULIN, to the chains. OTULIN has high affinity for Met1 chains, several fold higher than reported for NEMO, the prototypical Met1-Ub-binding protein (Keusekotten et al., 2013; Rahighi et al., 2009). Possibly, HOIL-1 or another factor with Met1-Ub-binding capacity (Haas et al., 2009) would prevent CYLD from gaining access to and cleaving the Met1-Ub on HOIP, whereas OTULIN would have access via its higher affinity toward Met1-Ub. However, further investigations are needed to uncover the basis of this differential role of CYLD and OTULIN in regulation of HOIP ubiquitination. It will also be of interest to investigate the possibility that HOIP ubiquitination could be an auto-regulatory mechanism controlled by OTULIN, for example, by inhibiting intrinsic LUBAC activity or affecting its recruitment to receptor complexes.

CYLD Regulation of LUBAC Substrates

An important insight from our study was that CYLD regulates the extension of Lys63 and Met1 linkages on RIPK2. Although depletion of CYLD resulted in extended Ub modifications on RIPK2 (and RIPK1 after TNF treatment), the full extent of regulation of RIPK2 ubiquitination by CYLD (and OTULIN) was uncovered only when we functionally inhibited IAP proteins. CYLD also regulated the ubiquitination of RIPK1, but inhibition of IAP proteins had a less dramatic effect. This suggests that deposition of Ub modifications at the NOD2 complex, the TNFR1 complex, and possibly other immune complexes is continuously coordinated through the opposing activities of Ub ligases and DUBs. This could enable dynamic alterations to the linkage composition of Ub chains within these signaling complexes. Such a mechanism is described for A20, which removes Lys63 linkages and assembles Lys48-Ub to terminate signaling (Wertz et al., 2004). In response to IL-1R stimulation, Met1 linkages are formed almost exclusively on existing Lys63-Ub conjugated to IRAK1, a component of the MyD88 signalosome (Emmerich et al., 2013). Analogously, the Ub-linkage composition of Ub-RIPK2 following NOD2 stimulation showed that Lys63-Ub is the first linkage type conjugated to RIPK2 whereas Met1 linkages are formed only on RIPK2 molecules that are already polyubiquitinated (Fiil et al., 2013). An intriguing possibility is that CYLD functions to trim Lys63-linked Ub chains to facilitate their Met1 ubiquitination by LUBAC.

Of note, during revision of this manuscript, a study by Draber et al. (2015) reported that OTULIN was not stably associated with receptor-signaling complexes and suggested that OTULIN does not regulate ubiquitination of RIPK2. However, the data presented here and in our previous study (Fiil et al., 2013) indicate that OTULIN has access to the endogenous NOD2 complex (irrespective of whether it associates stably or not), where it restricts RIPK2 ubiquitination alongside CYLD. The underlying reason for the discrepancy is not clear at this time but could be due to a difference in experimental approaches.

Functional Role of CYLD and Lys63-Ub

The functional requirement of Lys63-Ub for immune receptor signaling is controversial and appears to be specific to individual receptor systems (Ori et al., 2013; van Wijk et al., 2012; Xu et al., 2009). It was therefore important to establish whether or not K63-Ub contributes to NOD2 signaling. Using linkage-selective Ub binders to interfere with Lys63-Ub function together with silencing of the E2 Ubc13, we established that Lys63 linkages are essential for productive NOD2 signaling. Interestingly, the NOD2 pathway is equally dependent on LUBAC function and Met1-Ub (Damgaard et al., 2012; Warner et al., 2013), illustrating the non-redundant signaling properties of these linkages. In contrast, Lys63-Ub is reported to be largely dispensable in the

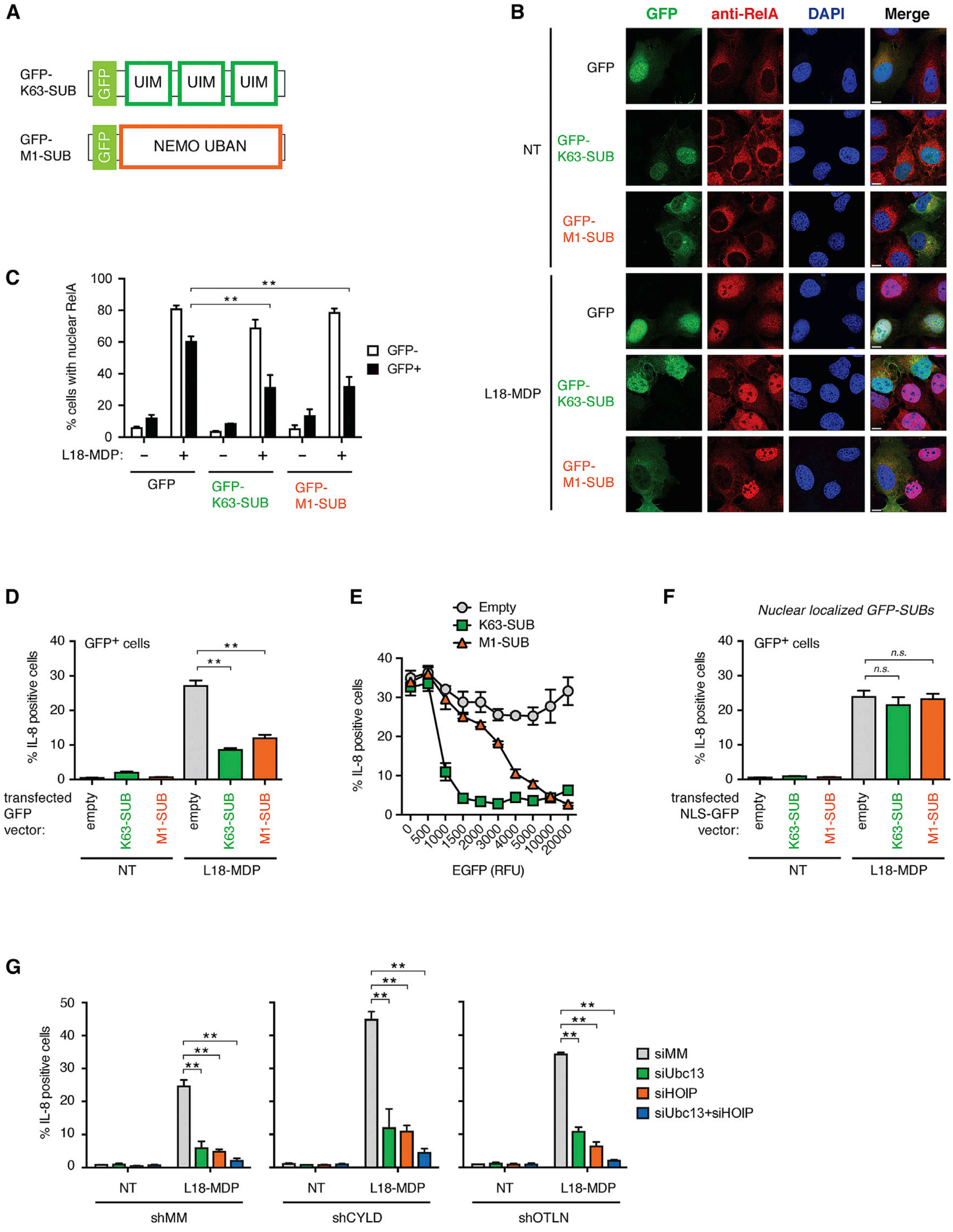

Figure 7. Lys63-Ub and Met1-Ub Are Individually Indispensable for NOD2 Signaling

(A) Schematic representation of the GFP-tagged Ub-binding constructs used for transient expression in cells (GFP-M1-SUB: UBAN domain from NEMO; GFP-K63-SUB: three UIMs from RAP80 in tandem).

(B) Immunofluorescence analysis of nuclear translocation of RelA (red) in response to L18-MDP stimulation (1 μg/ml; 1 hr) or no treatment (NT) in U2OS/NOD2 cells transfected with GFP, GFP-K63-SUB, or GFP-M1-SUB (green) for 24 hr. The scale bar represents 10 μm.

(C) Quantification of (B).

(D) Intracellular flow cytometry analysis of IL-8 in U2OS/NOD2 cells transfected as indicated for 48 hr before L18-MDP stimulation (200 ng/ml; 4 hr).

(legend continued on next page)

TNFR1 pathway (van Wijk et al., 2012; Xu et al., 2009), and although Met1-Ub contributes to pro-inflammatory signaling, it also has a prominent role in regulating the formation of cell death complexes (Gerlach et al., 2011; Ikeda et al., 2011; Keusekotten et al., 2013). The molecular basis underlying the receptor-specific functions of Lys63- and Met1-Ub is not understood, but it might reflect how ubiquitination is coordinated at receptor complexes. For example, the generation of Lys63/Met1 hybrid chains in the IL-1R pathway probably explains the exquisite dependency on the Lys63 linkage within the system (Emmerich et al., 2013).

Even though CYLD is well known to regulate inflammation and innate immune responses, its role in regulating the NOD2 pathway had not been defined. In line with the requirement of Lys63- and Met1-Ub in NOD2, but not TNFR1, signaling, we found that endogenous CYLD was critical for limiting productive NOD2 signaling but that it did not appreciably affect TNF signaling as judged by IL-8 production. Interestingly, CYLD is reported to negatively regulate the innate immune response to infection by *Listeria monocytogenes*, an intracellular bacterial pathogen recognized by NOD1 and NOD2 (Kim et al., 2008; Nishanth et al., 2013). This suggests that the regulation of RIPK2 ubiquitination by CYLD (and LUBAC and OTULIN) could influence the response to infection by intracellular bacteria.

In conclusion, our study exemplifies how ubiquitination following innate immune receptor activation is carefully controlled by LUBAC and its associated DUB activities CYLD and OTULIN for accurate regulation of downstream signaling and pro-inflammatory responses.

EXPERIMENTAL PROCEDURES

Purification of Endogenous Ub Conjugates

Ub conjugates were purified from cell lysates using affinity reagents TUBE, M1-SUB, and K63-SUB (Fiil et al., 2013; Thorslund et al., 2015) or UBA^{Ubq}. Briefly, cells were lysed in buffer containing 20 mM Na_2HPO_4, 20 mM NaH_2PO_4, 1% (v/v) NP-40, and 2 mM EDTA and supplemented with 5 mM N-ethylmaleimide, cOmplete protease inhibitors, and PhosSTOP (Roche). The affinity reagents TUBE (50 μg/ml), M1-SUB (100 μg/ml), K63-SUB (15 μg/ml), or UBA^{Ubq} (150 μg/ml) were either added directly to the cell lysates or pre-bound to Glutathione Sepharose 4B beads (GE Healthcare) for at least 1 hr. For K63-SUB pull-down, the Thermo Scientific Pierce Streptavidin Magnetic Beads were washed in lysis buffer and incubated with the biotinylated K63-SUB in the lysis buffer for at least 1 hr agitating at 4°C, followed by three washes. For all pull-downs, lysates were cleared by centrifugation, mixed with beads, and incubated agitating at 4°C for a minimum of 2 hr. The beads were washed four times in 500 μl of ice-cold PBS 0.1% (v/v) Tween-20 or TUBE lysis buffer. The bound material was eluted with 15 mM glutathione in PBS or with 1× sample loading buffer.

Immunofluorescence Staining and Confocal Microscopy

Low-density U2OS/NOD2 cell cultures grown on coverslips were transfected using Fugene6 (Promega) according to manufacturer's protocol. After 24 hr, the cells were stimulated with 1 μg/ml L18-MDP for 1 hr. The cells were fixed in 4% formaldehyde and permeabilized by 0.1% (v/v) Triton X-100 in PBS. Blocking was performed in 5% (w/v) BSA and 0.01% (v/v) Triton X-100 in PBS, and cells were stained with primary antibodies and fluorescently labeled secondary antibodies in the blocking solution for 1 hr at room temperature. The nuclei were stained with 1 μg/ml DAPI for 10 min. Coverslips were mounted on slides using ProLong Gold Antifade Reagent (Life Technologies). Images were acquired on a Carl Zeiss LSM710 confocal microscope equipped with 20× dry and 63× oil lenses. To quantify RelA translocation, images were semi-automatically processed in FiJi software using a macro. Briefly, nuclear staining (DAPI channel) was used to create a mask, which was used to measure fluorescence intensities in green (transfected cells) and red (RelA) channels.

Intracellular Flow Cytometry of IL-8

U2OS/NOD2 cells, transfected using Fugene6, were stimulated 24–48 hr after transfection with 200–300 ng/ml L18-MDP for 4–6 hr in the presence of 5 μg/ml Brefeldin A and 2 μM Monensin (BioLegend) protein transport inhibitors. After stimulation, cells were washed with PBS, dissociated by Trypsin/EDTA solution (GIBCO Life Technologies), and collected by centrifugation. Cells were fixed with IC Fixation Buffer (eBioscence) O/N at 4°C; washed with PBS; permeabilized using Perm/Wash Buffer containing 2% (v/v) FCS, 0.1% (w/v) saponin, and 0.1% (w/v) NaN_3 in PBS; and incubated in the Perm/Wash Buffer with anti-IL-8/allophycocyanin (APC) for 1 hr at room temperature. The cells were analyzed by FACS Canto Flow Cytometer (BD Bioscience) and data processed using FlowJo software (TreeStar). APC and GFP levels were acquired using 633 nm and 488 nm laser, respectively.

DUB Assays

Ub conjugates from L18-MDP-treated cells were isolated by M1-SUB (100 μg/ml) or GST-UBA^{Ubq} (150 μg/ml) pre-bound to GST beads as described under purification of endogenous Ub conjugates. After wash, beads were re-suspended in DUB buffer (50 mM HEPES [pH 7.5], 100 mM NaCl, 2 mM DTT, 1 mM $MnCl_2$, and 0.01% Brij-35). For siMM/shMM or siCYLD/shCYLD U2OS/NOD2 experiments, Ub conjugates incubated without or with DUBs (USP21 [0.5 μM], OTULIN [0.4–1 μM], OTULIN C/A [1 μM], vOTU [0.4 μM], OTUB1 [15 μM], OTUD1 [0.2 μM], and AMSH* [3 μM]). Samples were incubated for 15 min (Figure 6B) or 1 hr (Figure 6A). For the time course experiment, DUBs OTUD1 (0.2 μM) and OTULIN (0.4 μM) incubated with the Ub conjugates for 15, 60, or 240 min and USP21 (0.5 μM) and the no DUB control incubated for 60 min. For spiked in GST-Met1-Ub4 experiments, 4 μg was added to the Ub conjugates and incubated with DUBs (OTULIN [0.4 μM] and OTUD1 [0.2 μM]) or no DUB for 4 hr. For the THP1 cells, Ub conjugates incubated for 1 hr with or without DUBs OTULIN (1 μM), OTUD1 (1 μM), and Usp21 (0.5 μM). All samples incubated at 30°C with shaking, and loading sample buffer was added to stop the reaction.

Cytometric Bead Array

Secreted cytokines (Il-1β, IL-6, and TNF) were measured from culture supernatants from BMDC after 24 hr with or without MDP with the Cytometric Bead Array (CBA) (BD Biosciences) according to manufacturer's protocol.

Generation of Stable CRISPR/Cas9 HCT-116 Cells

To generate stable HCT-116 knockout cell lines, the cells were transfected using Fugene HD with the RNF31 CRISPR/Cas9 KO Plasmid (sc-412436; Santa Cruz Biotechnology) containing gRNA, Cas9, and EGFP marker. After 36 hr, top 10% of GFP-positive cells were sorted by flow cytometry and cloned by limiting dilution to obtain single-cell clones. Individual clones were validated by western blotting with HOIP-specific antibodies.

(E) Gating of cells in (D) based on GFP levels (relative fluorescence units [RFUs]). RFU values on x axis indicate the maximal RFU in each gate with the previous value defining the lower limit, except of the "zero" RFU population, which includes cells with values up to 100 RFUs.
(F) As in (D) except that GFP, GFP-K63-SUB, or GFP-M1-SUB contains a nuclear localization signal (NLS).
(G) Intracellular flow cytometry analysis of IL-8 in control (MM) U2OS/NOD2 cells or cells stably depleted for CYLD or OTULIN; depleted for HOIP, Ubc13, or both by siRNA as indicated; and treated with L18-MDP (200 ng/ml; 4 hr) or not treated (NT).
Data in (C)–(G) represent the mean ± SEM of at least three independent experiments, each performed in duplicate. **$p < 0.01$. See also Figure S7.

Statistical Analysis

Statistical analysis was performed in Using Prism 6 (GraphPad Software). The two-tailed Student's tests were used to determine statistical significance in Figures 1D, 3D, and 3E; at all other instances, two-way ANOVA was used to determine statistical significance.

SUPPLEMENTAL INFORMATION

Supplemental Information includes Supplemental Experimental Procedures and seven figures and can be found with this article online at http://dx.doi.org/10.1016/j.celrep.2016.02.062.

AUTHOR CONTRIBUTIONS

Conceptualization, M.G.-H.; Methodology, M.H. and B.K.F.; Investigation, M.H., B.K.F., D.L., M.Z., K.B., M.Y., and R.B.D.; Resources, P.R.E. and D.K.; Writing – Original Draft, M.G.-H., M.H., and B.K.F.; Writing – Review & Editing, M.G.-H., M.H., B.K.F., P.J.J., D.K., and R.B.D. Funding Acquisition, M.G.-H., B.K.F., P.J.J., and D.K.

ACKNOWLEDGMENTS

This work was supported by the Ludwig Institute for Cancer Research. M.G.-H. is supported by a Wellcome Trust Fellowship (102894/Z/13/Z), a Sapere Aude: Danish Council for independent Research Starting Grant, and the Lundbeck Foundation. B.K.F. is supported by a Carlsberg travel stipend. P.J.J. is supported by a Max Eder-Program grant from the Deutsche Krebshilfe (program no. 111738), a Human Frontiers Science Program grant (program no. RGY0073/2012), and a research grant from the Deutsche Forschungsgemeinschaft (FOR2036). P.J.J. and M.Y. are supported by an Else Kröner-Fresenius-Stiftung grant 2014_A185. Work in the D.K. lab is supported by the Medical Research Council (U105192732), the European Research Council (309756), and the Lister Institute for Preventive Medicine. M.G.-H. and D.K. are supported by the EMBO Young Investigator Programme. We thank Jonathan Pruneda for help with UbiCRest reagents, Niels Mailand and Tina Thorslund for plasmids, Tetralogic Pharmaceuticals for compound A, and members of the group for helpful suggestions and reading the manuscript.

Received: November 16, 2015
Revised: January 28, 2016
Accepted: February 12, 2016
Published: March 17, 2016

REFERENCES

Abbott, D.W., Wilkins, A., Asara, J.M., and Cantley, L.C. (2004). The Crohn's disease protein, NOD2, requires RIP2 in order to induce ubiquitinylation of a novel site on NEMO. Curr. Biol. *14*, 2217–2227.

Bauler, L.D., Duckett, C.S., and O'Riordan, M.X. (2008). XIAP regulates cytosol-specific innate immunity to Listeria infection. PLoS Pathog. *4*, e1000142.

Bertrand, M.J., Doiron, K., Labbé, K., Korneluk, R.G., Barker, P.A., and Saleh, M. (2009). Cellular inhibitors of apoptosis cIAP1 and cIAP2 are required for innate immunity signaling by the pattern recognition receptors NOD1 and NOD2. Immunity *30*, 789–801.

Bignell, G.R., Warren, W., Seal, S., Takahashi, M., Rapley, E., Barfoot, R., Green, H., Brown, C., Biggs, P.J., Lakhani, S.R., et al. (2000). Identification of the familial cylindromatosis tumour-suppressor gene. Nat. Genet. *25*, 160–165.

Boisson, B., Laplantine, E., Prando, C., Giliani, S., Israelsson, E., Xu, Z., Abhyankar, A., Israël, L., Trevejo-Nunez, G., Bogunovic, D., et al. (2012). Immunodeficiency, autoinflammation and amylopectinosis in humans with inherited HOIL-1 and LUBAC deficiency. Nat. Immunol. *13*, 1178–1186.

Boisson, B., Laplantine, E., Dobbs, K., Cobat, A., Tarantino, N., Hazen, M., Lidov, H.G., Hopkins, G., Du, L., Belkadi, A., et al. (2015). Human HOIP and LUBAC deficiency underlies autoinflammation, immunodeficiency, amylopectinosis, and lymphangiectasia. J. Exp. Med. *212*, 939–951.

Brummelkamp, T.R., Nijman, S.M., Dirac, A.M., and Bernards, R. (2003). Loss of the cylindromatosis tumour suppressor inhibits apoptosis by activating NF-kappaB. Nature *424*, 797–801.

Damgaard, R.B., Nachbur, U., Yabal, M., Wong, W.W., Fiil, B.K., Kastirr, M., Rieser, E., Rickard, J.A., Bankovacki, A., Peschel, C., et al. (2012). The ubiquitin ligase XIAP recruits LUBAC for NOD2 signaling in inflammation and innate immunity. Mol. Cell *46*, 746–758.

Damgaard, R.B., Fiil, B.K., Speckmann, C., Yabal, M., zur Stadt, U., Bekker-Jensen, S., Jost, P.J., Ehl, S., Mailand, N., and Gyrd-Hansen, M. (2013). Disease-causing mutations in the XIAP BIR2 domain impair NOD2-dependent immune signalling. EMBO Mol. Med. *5*, 1278–1295.

Deng, L., Wang, C., Spencer, E., Yang, L., Braun, A., You, J., Slaughter, C., Pickart, C., and Chen, Z.J. (2000). Activation of the IkappaB kinase complex by TRAF6 requires a dimeric ubiquitin-conjugating enzyme complex and a unique polyubiquitin chain. Cell *103*, 351–361.

Draber, P., Kupka, S., Reichert, M., Draberova, H., Lafont, E., de Miguel, D., Spilgies, L., Surinova, S., Taraborrelli, L., Hartwig, T., et al. (2015). LUBAC-recruited CYLD and A20 regulate gene activation and cell death by exerting opposing effects on linear ubiquitin in signaling complexes. Cell Rep. *13*, 2258–2272.

Elliott, P.R., Nielsen, S.V., Marco-Casanova, P., Fiil, B.K., Keusekotten, K., Mailand, N., Freund, S.M., Gyrd-Hansen, M., and Komander, D. (2014). Molecular basis and regulation of OTULIN-LUBAC interaction. Mol. Cell *54*, 335–348.

Emmerich, C.H., Ordureau, A., Strickson, S., Arthur, J.S., Pedrioli, P.G., Komander, D., and Cohen, P. (2013). Activation of the canonical IKK complex by K63/M1-linked hybrid ubiquitin chains. Proc. Natl. Acad. Sci. USA *110*, 15247–15252.

Fiil, B.K., and Gyrd-Hansen, M. (2014). Met1-linked ubiquitination in immune signalling. FEBS J. *281*, 4337–4350.

Fiil, B.K., Damgaard, R.B., Wagner, S.A., Keusekotten, K., Fritsch, M., Bekker-Jensen, S., Mailand, N., Choudhary, C., Komander, D., and Gyrd-Hansen, M. (2013). OTULIN restricts Met1-linked ubiquitination to control innate immune signaling. Mol. Cell *50*, 818–830.

Gerlach, B., Cordier, S.M., Schmukle, A.C., Emmerich, C.H., Rieser, E., Haas, T.L., Webb, A.I., Rickard, J.A., Anderton, H., Wong, W.W., et al. (2011). Linear ubiquitination prevents inflammation and regulates immune signalling. Nature *471*, 591–596.

Haas, T.L., Emmerich, C.H., Gerlach, B., Schmukle, A.C., Cordier, S.M., Rieser, E., Feltham, R., Vince, J., Warnken, U., Wenger, T., et al. (2009). Recruitment of the linear ubiquitin chain assembly complex stabilizes the TNF-R1 signaling complex and is required for TNF-mediated gene induction. Mol. Cell *36*, 831–844.

Harhaj, E.W., and Dixit, V.M. (2012). Regulation of NF-κB by deubiquitinases. Immunol. Rev. *246*, 107–124.

Hasegawa, M., Fujimoto, Y., Lucas, P.C., Nakano, H., Fukase, K., Núñez, G., and Inohara, N. (2008). A critical role of RICK/RIP2 polyubiquitination in Nod-induced NF-kappaB activation. EMBO J. *27*, 373–383.

Hospenthal, M.K., Mevissen, T.E., and Komander, D. (2015). Deubiquitinase-based analysis of ubiquitin chain architecture using Ubiquitin Chain Restriction (UbiCRest). Nat. Protoc. *10*, 349–361.

Ikeda, F., Deribe, Y.L., Skånland, S.S., Stieglitz, B., Grabbe, C., Franz-Wachtel, M., van Wijk, S.J., Goswami, P., Nagy, V., Terzic, J., et al. (2011). SHARPIN forms a linear ubiquitin ligase complex regulating NF-κB activity and apoptosis. Nature *471*, 637–641.

Jiang, X., and Chen, Z.J. (2012). The role of ubiquitylation in immune defence and pathogen evasion. Nat. Rev. Immunol. *12*, 35–48.

Jono, H., Lim, J.H., Chen, L.F., Xu, H., Trompouki, E., Pan, Z.K., Mosialos, G., and Li, J.D. (2004). NF-kappaB is essential for induction of CYLD, the negative regulator of NF-kappaB: evidence for a novel inducible autoregulatory feedback pathway. J. Biol. Chem. *279*, 36171–36174.

Keusekotten, K., Elliott, P.R., Glockner, L., Fiil, B.K., Damgaard, R.B., Kulathu, Y., Wauer, T., Hospenthal, M.K., Gyrd-Hansen, M., Krappmann, D., et al. (2013). OTULIN antagonizes LUBAC signaling by specifically hydrolyzing Met1-linked polyubiquitin. Cell *153*, 1312–1326.

Kim, Y.G., Park, J.H., Shaw, M.H., Franchi, L., Inohara, N., and Núñez, G. (2008). The cytosolic sensors Nod1 and Nod2 are critical for bacterial recognition and host defense after exposure to Toll-like receptor ligands. Immunity *28*, 246–257.

Kirisako, T., Kamei, K., Murata, S., Kato, M., Fukumoto, H., Kanie, M., Sano, S., Tokunaga, F., Tanaka, K., and Iwai, K. (2006). A ubiquitin ligase complex assembles linear polyubiquitin chains. EMBO J. *25*, 4877–4887.

Komander, D., Reyes-Turcu, F., Licchesi, J.D., Odenwaelder, P., Wilkinson, K.D., and Barford, D. (2009). Molecular discrimination of structurally equivalent Lys 63-linked and linear polyubiquitin chains. EMBO Rep. *10*, 466–473.

Kovalenko, A., Chable-Bessia, C., Cantarella, G., Israël, A., Wallach, D., and Courtois, G. (2003). The tumour suppressor CYLD negatively regulates NF-kappaB signalling by deubiquitination. Nature *424*, 801–805.

Lee, E.G., Boone, D.L., Chai, S., Libby, S.L., Chien, M., Lodolce, J.P., and Ma, A. (2000). Failure to regulate TNF-induced NF-kappaB and cell death responses in A20-deficient mice. Science *289*, 2350–2354.

Michel, M.A., Elliott, P.R., Swatek, K.N., Simicek, M., Pruneda, J.N., Wagstaff, J.L., Freund, S.M., and Komander, D. (2015). Assembly and specific recognition of K29- and K33-linked polyubiquitin. Mol. Cell *58*, 95–109.

Nishanth, G., Deckert, M., Wex, K., Massoumi, R., Schweitzer, K., Naumann, M., and Schlüter, D. (2013). CYLD enhances severe listeriosis by impairing IL-6/STAT3-dependent fibrin production. PLoS Pathog. *9*, e1003455.

Ori, D., Kato, H., Sanjo, H., Tartey, S., Mino, T., Akira, S., and Takeuchi, O. (2013). Essential roles of K63-linked polyubiquitin-binding proteins TAB2 and TAB3 in B cell activation via MAPKs. J. Immunol. *190*, 4037–4045.

Philpott, D.J., Sorbara, M.T., Robertson, S.J., Croitoru, K., and Girardin, S.E. (2014). NOD proteins: regulators of inflammation in health and disease. Nat. Rev. Immunol. *14*, 9–23.

Rahighi, S., Ikeda, F., Kawasaki, M., Akutsu, M., Suzuki, N., Kato, R., Kensche, T., Uejima, T., Bloor, S., Komander, D., et al. (2009). Specific recognition of linear ubiquitin chains by NEMO is important for NF-kappaB activation. Cell *136*, 1098–1109.

Ritorto, M.S., Ewan, R., Perez-Oliva, A.B., Knebel, A., Buhrlage, S.J., Wightman, M., Kelly, S.M., Wood, N.T., Virdee, S., Gray, N.S., et al. (2014). Screening of DUB activity and specificity by MALDI-TOF mass spectrometry. Nat. Commun. *5*, 4763.

Rivkin, E., Almeida, S.M., Ceccarelli, D.F., Juang, Y.C., MacLean, T.A., Srikumar, T., Huang, H., Dunham, W.H., Fukumura, R., Xie, G., et al. (2013). The linear ubiquitin-specific deubiquitinase gumby regulates angiogenesis. Nature *498*, 318–324.

Sato, Y., Goto, E., Shibata, Y., Kubota, Y., Yamagata, A., Goto-Ito, S., Kubota, K., Inoue, J., Takekawa, M., Tokunaga, F., and Fukai, S. (2015). Structures of CYLD USP with Met1- or Lys63-linked diubiquitin reveal mechanisms for dual specificity. Nat. Struct. Mol. Biol. *22*, 222–229.

Schaeffer, V., Akutsu, M., Olma, M.H., Gomes, L.C., Kawasaki, M., and Dikic, I. (2014). Binding of OTULIN to the PUB domain of HOIP controls NF-κB signaling. Mol. Cell *54*, 349–361.

Sims, J.J., Scavone, F., Cooper, E.M., Kane, L.A., Youle, R.J., Boeke, J.D., and Cohen, R.E. (2012). Polyubiquitin-sensor proteins reveal localization and linkage-type dependence of cellular ubiquitin signaling. Nat. Methods *9*, 303–309.

Takiuchi, T., Nakagawa, T., Tamiya, H., Fujita, H., Sasaki, Y., Saeki, Y., Takeda, H., Sawasaki, T., Buchberger, A., Kimura, T., et al. (2014). Suppression of LUBAC-mediated linear ubiquitination by a specific interaction between LUBAC and the deubiquitinases CYLD and OTULIN. Genes to Cells *19*, 254–272.

Tang, J., Qu, L.K., Zhang, J., Wang, W., Michaelson, J.S., Degenhardt, Y.Y., El-Deiry, W.S., and Yang, X. (2006). Critical role for Daxx in regulating Mdm2. Nat. Cell Biol. *8*, 855–862.

Tao, M., Scacheri, P.C., Marinis, J.M., Harhaj, E.W., Matesic, L.E., and Abbott, D.W. (2009). ITCH K63-ubiquitinates the NOD2 binding protein, RIP2, to influence inflammatory signaling pathways. Curr. Biol. *19*, 1255–1263.

Thorslund, T., Ripplinger, A., Hoffmann, S., Wild, T., Uckelmann, M., Villumsen, B., Narita, T., Sixma, T.K., Choudhary, C., Bekker-Jensen, S., and Mailand, N. (2015). Histone H1 couples initiation and amplification of ubiquitin signalling after DNA damage. Nature *527*, 389–393.

Tokunaga, F., Nakagawa, T., Nakahara, M., Saeki, Y., Taniguchi, M., Sakata, S., Tanaka, K., Nakano, H., and Iwai, K. (2011). SHARPIN is a component of the NF-κB-activating linear ubiquitin chain assembly complex. Nature *471*, 633–636.

Trompouki, E., Hatzivassiliou, E., Tsichritzis, T., Farmer, H., Ashworth, A., and Mosialos, G. (2003). CYLD is a deubiquitinating enzyme that negatively regulates NF-kappaB activation by TNFR family members. Nature *424*, 793–796.

van Wijk, S.J., Fiskin, E., Putyrski, M., Pampaloni, F., Hou, J., Wild, P., Kensche, T., Grecco, H.E., Bastiaens, P., and Dikic, I. (2012). Fluorescence-based sensors to monitor localization and functions of linear and K63-linked ubiquitin chains in cells. Mol. Cell *47*, 797–809.

Warner, N., Burberry, A., Franchi, L., Kim, Y.G., McDonald, C., Sartor, M.A., and Núñez, G. (2013). A genome-wide siRNA screen reveals positive and negative regulators of the NOD2 and NF-κB signaling pathways. Sci. Signal. *6*, rs3.

Watanabe, T., Asano, N., Meng, G., Yamashita, K., Arai, Y., Sakurai, T., Kudo, M., Fuss, I.J., Kitani, A., Shimosegawa, T., et al. (2014). NOD2 downregulates colonic inflammation by IRF4-mediated inhibition of K63-linked polyubiquitination of RICK and TRAF6. Mucosal Immunol. *7*, 1312–1325.

Wertz, I.E., O'Rourke, K.M., Zhou, H., Eby, M., Aravind, L., Seshagiri, S., Wu, P., Wiesmann, C., Baker, R., Boone, D.L., et al. (2004). De-ubiquitination and ubiquitin ligase domains of A20 downregulate NF-kappaB signalling. Nature *430*, 694–699.

Xu, M., Skaug, B., Zeng, W., and Chen, Z.J. (2009). A ubiquitin replacement strategy in human cells reveals distinct mechanisms of IKK activation by TNFalpha and IL-1beta. Mol. Cell *36*, 302–314.

Yang, S., Wang, B., Humphries, F., Jackson, R., Healy, M.E., Bergin, R., Aviello, G., Hall, B., McNamara, D., Darby, T., et al. (2013). Pellino3 ubiquitinates RIP2 and mediates Nod2-induced signaling and protective effects in colitis. Nat. Immunol. *14*, 927–936.

Cell Reports, Volume *14*

Supplemental Information

CYLD Limits Lys63- and Met1-Linked Ubiquitin at Receptor Complexes to Regulate Innate Immune Signaling

Matous Hrdinka, Berthe Katrine Fiil, Mattia Zucca, Derek Leske, Katrin Bagola, Monica Yabal, Paul R. Elliott, Rune Busk Damgaard, David Komander, Philipp J. Jost, and Mads Gyrd-Hansen

Supplemental Information

Supplemental Experimental Procedures

Plasmids and cloning

The plasmids used in this study were obtained from the following sources or generated as described bellow: pcDNA3-HOIP^{WT}-V5/His, pcDNA3-HOIP^{mutRx2} -V5/His, and pcDNA3-HOIL1-V5/His were a kind gift from Prof. Henning Walczak (University College London, London, UK). The mutated HOIP constructs pcDNA3-HOIP$^{PUB+NZF}$-V5/His and pcDNA3-HOIP^{N102D}-V5/His were described previously (Elliott et al., 2014). The OTULIN constructs pcDNA3-HA-OTULIN^{WT}, pcDNA3-HA-OTULIN^{C129A}, and pcDNA3-3xHA-Ub-AVPI-4xUbGS were described previously (Fiil et al., 2013). The pGEX-6P-1-Ubiquitin4x construct was subcloned from a plasmid kindly provided by Prof. Ivan Dikic (Goethe University, Frankfurt am Main, Germany). The NF-κB luciferase reporter plasmids pBIIX-Luc and TK-renilla-Luc and the XIAP constructs pcDNA3-3xHA-XIAP^{WT}, pcDNA3-3xHA-XIAP^{F495A} have been described previously (Damgaard et al., 2012; Gyrd-Hansen et al., 2008). The pCMV-2-FLAG-CYLD^{WT} was cloned from human cDNA and the catalytically inactive mutant FLAG-CYLD^{C601A} was generated by PCR-based site-directed mutagenesis. The constructs for ubiquitin binding pGEX-6P-1-NEMO-CoZi1-His6 (M1-SUB) and pEGFP-C1-NEMO-CoZi (GFP-M1-SUB) and pGEX-6P-1-Ubiquillin-UBA4x (TUBE) were described previously (Fiil et al., 2013). The pGEX-6P-1-Ubiquillin-UBA1x construct consisting of a single UBA domain was generated by PCR. The constructs for Lys63-linked polyubiquitin binder pET104-DEST-RAP80-UIM3x (K63-SUB) was a kind gift from Niels Mailand and Tina Thorslund and was cloned into pcDNA-DEST53-RAP80-UIM3x to make GFP-K63-SUB. To create GFP-K63-SUB, GFP-M1-SUB and GFP alone targeted to the nucleus, the SV40 nuclear localization sequence (NLS, sequence: PKKKRKV) was inserted between GFP and the Ub binder. Full sequences will be made available upon request.

Antibodies and immunoprecipitation reagents

The following antibodies and reagents were used according to the manufacturers' instructions: rat monoclonal anti-HA (Roche Diagnostics, Burgess Hill, UK), mouse monoclonal anti-JNK1/2 and anti-phospho-JNK (Tyr183/Tyr185) (BD Biosciences, San Jose, CA), rabbit monoclonal anti-JNK, ERK1/2 and phospho-ERK1/2 (Cell Signaling Technology, Danvers, MA, Figure S3B), mouse monoclonal anti-

XIAP (Abcam, Cambridge, MA), mouse monoclonal anti-ubiquitin (Imgenex, San Diego, CA, or P4D1, Cell Signaling Technology), rabbit monoclonal Lys63 antibody (Millipore), rabbit monoclonal anti-RIP1 (D94C12), rabbit polyclonal and mouse monoclonal anti-RIPK2 (Santa Cruz Biotechnology, Santa Cruz, CA), mouse monoclonal anti-RIPK2 (Figure S3B, BD Biosciences), rabbit polyclonal and mouse monoclonal anti-NEMO/IKKγ (Santa Cruz Biotechnology and BD Biosciences, respectively), and rabbit polyclonal anti-SHARPIN (ProteinTech, Chicago, IL), rabbit polyclonal anti-RBCK1/HOIL-1 (Novus Biologicals, Littleton, CO). Rabbit polyclonal anti-HOIP/RNF31 (Sigma-Aldrich, Gillingham, UK or Bethyl Laboratories, Montgomery, USA). Mouse monoclonal anti-V5 (AbD Serotec, Kidlington, UK), monoclonal anti-β-actin (Chemicon Millipore, Billerica, MA), rabbit polyclonal antibodies to RelA/p65, p38 MAP Kinase and IκBα, and rabbit polyclonal antibodies to phosphorylated p38 MAP Kinase (Thr180/Tyr182), RelA/p65 (Ser536) and IκBα (Ser32) (Cell Signaling Technology), rabbit polyclonal anti-Fam105B/OTULIN (Abcam), mouse monoclonal and rabbit polyclonal anti-CYLD (Santa Cruz Biotechnology, and Cell Signaling Technology, respectively), rabbit monoclonal anti-A20/TNFAIP3 (D13H3, Cell Signaling Technology), rabbit polyclonal anti-Ubc13 (Cell Signaling Technology), and rat monoclonal anti-cIAP1 (Enzo Life Sciences, Exeter, UK). The HRP-conjugated secondary antibodies were purchased from Bio-Rad (anti-rabbit) and Dako (anti-mouse). For immunofluorescence, primary antibodies rabbit polyclonal anti-RelA (Santa Cruz Biotechnology) or rabbit monoclonal anti-NF-κB p65 (D14E12, Cell Signaling), mouse monoclonal anti-FLAG M2 (Sigma-Aldrich) and secondary antibodies anti-mouse IgG / Alexa Fluor 488 and anti-rabbit IgG/Alexa Fluor 568 (Invitrogen Life Technologies) were used. For intracellular staining of IL-8 for flow cytometry the APC conjugated mouse anti-human IL-8 Antibody (clone E8N1, BioLegend, SanDiego, CA) was used. For immunoprecipitation anti-HA-agarose conjugate (Clone HA-7, A2095), anti-FLAG affinity gel (Clone M2, A2220) and anti-V5 affinity gel (Clone V5-10, A7345) were from Sigma-Aldrich.

Cell lines

NOD2-expressing U2OS-Flp-In™ T-REx™ (U2OS/NOD2) cells were cultured in DMEM GlutaMax (Gibco Life Technologies) supplemented with 10 % (v/v) FBS (Sigma-Aldrich) and 1 % (v/v) Penicillin-Streptomycin (Gibco Life Technologies) and transfected using Fugene 6 (Promega). Throughout the study, the U2OS/NOD2 cells were cultured and stimulated in the absence of doxycycline unless otherwise indicated. HEK293T (Figure 3E, Suppl. Figure S3D) and HEK293FT were cultured in DMEM

GlutaMax (Gibco Life Technologies) supplemented with 10 % (v/v) FBS (Sigma-Aldrich) and 1 % (v/v) Penicillin-Streptomycin (Gibco Life Technologies) and transfected using Fugene HD (Promega). THP1 cells were maintained at density not exceeding 1x10^6 cells per ml of culture media RPMI1640 GlutaMax (Invitrogen) supplemented with 10 % (v/v) FBS (Sigma-Aldrich), 1 % (v/v) Penicillin-Streptomycin (Gibco Life Technologies), 1 mM sodium pyruvate (Gibco Life Technologies), and 50 μM 2-mercaptoethanol (Lonza). The HCT-116 cells were cultured in McCoy's GlutaMax medium (Gibco Life Technologies) supplemented with 10 % (v/v) FBS (Sigma-Aldrich) and 1 % (v/v) Penicillin-Streptomycin (Gibco Life Technologies).

BMDC generation

For generation of bone marrow derived dendritic cells (BMDC), the bone marrow from WT and *Cyld*$^{-/-}$ mice was cultured in VLE-RPMI-medium (Biochrom Merck Millipore) supplemented with 10 % Sera plus FBS (PAN Biotech), Glutamine, Penicillin-Streptomycin, β-mercapto-ethanol (Gibco), and 20 nM GM-CSF (Peprotech). Culture medium was supplemented on day 3 and day 6 and experiments were performed on day 7. *Cyld*$^{-/-}$ mice were obtained from Prof. Dr. Dirk Schlüter, Otto-von-Guericke-University Magdeburg, Germany.

Luciferase reporter assays

HEK293FT, HCT-116 WT or HCT-116 HOIP KO cells were co-transfected with the NF-κB luciferase reporter construct pBIIX-luc and a thymidine kinase-renilla luciferase construct for normalization of transfection efficiency. HEK293FT and HCT-116 cells were co-transfected with additional plasmids as indicated elsewhere and assays were performed as previously described (Damgaard et al., 2012). Individual experiments were performed in duplicate.

Immunoprecipitation

Immunoprecipitation of HA-NOD2, FLAG-CYLD, endogenous SHARPIN, or HOIP-V5 from U2OS/NOD2 or HEK293T/FT cells. Cells were transfected and treated as indicated. Cells were lysed in the IP buffer (25 mM HEPES pH 7.4, 150 mM KCl, 2 mM $MgCl_2$, 1 mM EGTA, 0.5 % (v/v) Triton X-100) supplemented with 5 mM N-Ethylmaleimide (NEM; Sigma Aldrich), cOmplete protease inhibitor cocktail and PhosSTOP (Roche) for 30 min on ice. Lysates were cleared by centrifugation and were

incubated at 4°C overnight with anti-HA (Sigma Aldrich), anti-FLAG affinity gel (Sigma Aldrich), anti SHARPIN (ProteinTech), or anti-V5-coupled beads (Sigma Aldrich). Beads were washed four times in 500 µl of ice-cold IP buffer and bound material eluted with 0.2 M glycine pH 2.5.

TNF receptor pull-down

U2OS/NOD2 cells were treated with FLAG-TNF (Enzo, 100 ng/ml) for indicated time points. Cells were lysed in buffer containing: 30 mM TrisHCl, 120 mM NaCl, 2 mM EDTA, 2 mM KCl, 1 % Triton X-100 supplemented with 1 mM DTT, 5 mM N-Ethylmaleimide (NEM), cOmplete protease inhibitors, and phosSTOP (Roche). Lysates were incubated with anti-FLAG-agarose beads over night at 4°C. The bound material was eluted with 100 µg/ml FLAG peptide (Sigma Aldrich).

Receptor stimulation

Cells were treated with NOD2 ligand L18-MDP (InvivoGen) or a recombinant TNF receptor ligand TNF-α (Peprotech, Rocky Hill, NJ) added directly to the culture medium for the times indicated. Cells were lysed in TUBE lysis buffer (20 mM Na_2HPO_4, 20 mM NaH_2PO_4, 1 % NP-40, 2 mM EDTA) supplemented with 5 mM N-Ethylmaleimide (NEM; Sigma Aldrich), cOmplete protease inhibitor cocktail and PhosSTOP and cleared by centrifugation before lysis loading sample buffer (LSB) was added. BMDC were stimulated with 10 μg/ml MDP (Invivogen) in the absence of GM-CSF and for BMDCs time course, cells were harvested, washed in PBS and lysed in RIPA buffer supplemented with cOmplete Protease Inhibitor Cocktail (Roche) and the phosphatase inhibitors sodium orthovanadate and sodium flouride (Sigma-Aldrich).

RNA isolation, cDNA synthesis and qPCR

Total RNA was isolated from cells using RNeasy Mini Kit (Qiagen) and DNase digestion was performed on-column with the RNase-Free DNase Set (Qiagen) according to manufacturer's protocol. Total RNA was reverse transcribed with RevertAid™ Reverse Transcriptase (Thermo-Fisher Scientific) and a mixture of anchored oligo(dT)$_{20}$ primers and random pentadecamers in the presence of RiboLock (Thermo-Fisher Scientific). qPCR was performed using SYBR Select Master Mix (Applied Biosystems).

cDNA was amplified with the following primer pairs:

Hypoxanthine phosphoribosyltransferase (HPRT; used as reference gene for normalization):

5’-AGCCAGACTTTGTTGGATTTG-3’ and 5’-TTTACTGGCGATGTCAATAGG-3’,

TNF: 5’-TGCTGCAGGACTTGAGAAGA-3’ and 5’-GAGGAAGGCCTAAGGTCCAC-3’,

IL-8: 5’-TCTGGCAACCCTAGTCTGCT-3’ and 5’-AAACCAAGGCACAGTGGAAC -3’,

OTULIN: 5’-ACATGAAAGAGGGGCATCAG-3’ and 5’-TTCATACACGTTGCTTTCTGTGT-3’

CYLD: 5’-TTTGCGTGTGTTGAAAGTACAAT-3’ and 5’-TTCCTGCGTCACACTCTCTG-3’

A20: 5’-ATGCACCGATACACACTGGA-3’ and 5’-GGATGATCTCCCGAAACTGA-3’

All experiments were performed three times with two technical replicates per experiment.

Transient and stable RNAi knock-down

The cells were reverse transfected with siRNA oligonucleotides (final concentration 35 nM siRNA oligo) using Lipofectamine RNAiMAX (Invitrogen Life Technologies) according to the manufacturer’s instructions. The following siRNA oligonucleotides (Sigma-Aldrich) were used for RNAi-mediated knockdown of OTULIN: SASI_Hs01_00040471, CAAAUGAGGCGGAGGAAUA[dT][dT]; CYLD: SASI_Hs02_00309209, GAACAGAUUCCACUCUUUA[dT][dT], Ubc13 #1: SASI_Hs01_00012964, CAGACAUCUUCAGUCCUUU[dT][dT]; Ubc13 #2: SASI_Hs01_00012965, GAAGAAUAUGUUUAGAUAU[dT][dT]; HOIP: GGCGUGGUGUCAAGUUUAA[dT][dT] (Haas et al., 2009); Mismatched control (siMM): Mission siNEG Ctrl 1 (Cat. #SIC001).

To generate stable knock-down of CYLD (shCYLD) and mismatch control knock-down (shMM) in U2OS/NOD2, THP1 and HCT-116 cells the validated siRNA oligos were cloned into pSUPER (BglII/HindIII) vector as 60-bp hairpins. shCYLD: 5'-GATCCCCGAACAGATTCCA CTCTTTATTCAAGAGATAAAGAGTGGAATCTGTTCTTTTTA-3', shMM: 5'-GATCCCCTTCTC CGAACGTGTCACGTTTCAAGAGAACGTGACACGTTCGGAGAATTTTTA-3'

The hairpins were then subcloned into pTRIP-GFP (Watabe-Uchida et al., 2006) or pTRIP-PURO lentiviral vectors via EcoRI site and validated by sequencing. The shRNA vector TRC2-pLKO-PURO for stable OTULIN knock-down (shOTLN) was purchased from Sigma-Aldrich (Cat. #TRCN0000285391). The lentiviral particles were generated in HEK293FT cells by cotransfection of the shRNA vectors with packaging vectors psPAX2 and pMD.G. The virus-containing supernatants were harvested after 72 h, filtered through 0.45 μm filters, and concentrated by precipitation with PEG-

8000. The cells were infected with the concentrated lentiviral particles in the presence of 6-10 μg/ml polybrene (Sigma-Aldrich) over night and selected with 1 μg/ml puromycin for one week.

Expression and purification of ubiquitin binding entities for pull down

E. coli BL21 cells were transfected with plasmids encoding C-terminally His6-tagged ubiquitin binding domains. For the expression and *in vivo* biotinylation of the K63-SUB, BL21 cells expressing the BirA biotin ligase from a pCDF-Duet vector were used. Over night cultures were diluted 1:100 into TB medium supplemented with appropriate antibiotics. Cells were grown at 37°C for 6 h, cooled down to 16°C, protein expression was induced with 500 *μ*M IPTG followed by incubation for 17 h at 16°C and 180 rpm before harvesting. The cells were lysed in His6-binding buffer (50 mM Na-phosphate pH 7.5, 300 mM NaCl, 10 % Glycerol, 10 mM Imidazole, 0.5 mM TCEP) using a high-pressure homogenizer. Lysate was cleared and loaded on a FPLC for protein purification via a 5 ml HisTrap FF column. After injecting the sample, the column was washed with ten column volumes of 50 mM Na-phosphate pH 7.5, 300 mM NaCl, 10 % Glycerol, 30 mM Imidazole, 0.5 mM TCEP followed by protein elution with 50 mM Na-phosphate pH 7.5 , 300 mM NaCl, 10 % Glycerol, 300 or 500 mM Imidazole, 0.5 mM TCEP. Subsequent dialysis of protein solution was performed in 2-3 steps in 50 mM Na-phosphate pH 7.5, 150 mM NaCl, 10 % Glycerol, 0.5 mM TCEP. Proteins were frozen in liquid nitrogen and stored at -80°C.

Expression and purification of GST-Ubiquitin4x

E coli BL21 Rosetta chemically competent cells were transfected with the GST-Ubiquitin4x construct and grown over night in LB, 10 % TB, Ampicillin, shaking at 37°C. Cells were diluted 1:10 in 1 l of LB, 10 % TB, Ampicillin and grown at 37°C until $OD_{(600)}$ = 0.6. Cells were cooled down on ice, protein expression was induced by adding 0.1 mM IPTG and grown over night at 18°C before harvesting. Lysis of cells was performed in PBS with 300 mM NaCl, 1 mM DTT, cOmplete protease inhibitor mix (Roche) by sonication. Lysate was cleared by centrifugation and incubated for 2 h with equilibrated Glutathione Sepharose™ 4 Fast Flow, rotating at 4°C. Resin was washed with TBS before elution of the purified proteins using (50 mM Tris-HCl pH 8.5, 150 mM NaCl, 10 % glycerol, 2 mM β-mercaptoethanol, 20 mM reduced glutathione). Proteins were stored at -80°C in TBS, 10 % Glycerol, and 1 mM DTT.

Supplemental Figure Legends

Figure S1. CYLD antagonizes LUBAC function but does not affect HOIP ubiquitination. Data are associated with Figure 1.

(A) HEK293FT cell lysates from experiment shown in Figure 1D were analyzed by immunoblotting as indicated. (B) *In vitro* DUB assay on Lys63 or GST-Met1 tetraUb with CYLD WT and catalytically inactive (C601A) isolated via their FLAG-tag followed by immunoblotting or coomassie staining to determine level of purified CYLD. (C) Immunoblot for Ub from experiment shown in Figure 1E of purified Ub-conjugates using the UBA^{Ubq}, M1-SUB or K63-SUB from control U2OS/NOD2 cells or cells depleted for CYLD (shCYLD) or OTULIN (shOTLN) as indicated after treatment with L18-MDP (200 ng/ml, 1 h) or TNF (10 ng/ml, 10 min). (D) and (E) Immunoblot for Ub in purified Ub-conjugates using the M1-SUB from THP1 cells (D) from the experiment shown in Figure 1F or HCT-116 cells (E) from the experiment shown in Figure 1G. (F) Purified Ub conjugates using M1-SUB and TUBE from HEK293FT from the experiment shown in Figure 1H were analyzed by immunoblotting as indicated. Asterisk denotes unspecific band detected by the antibody.

Figure S2. CYLD restricts NOD2 signaling and cytokine production. Data are associated with Figure 2.

(A) Data from Figure 2D plotted as mean fluorescence intensity (MFI) of all cells in the IL-8/APC channel. (B) Immunoblotting of key NF-κB and MAPK signaling proteins in U2OS/NOD2 control, OTULIN and/or CYLD-depleted cells upon L18-MDP treatment (5 ng/ml or 200 ng/ml for 1 h and 2 h). (C) NF-κB activity in HCT-116 WT or HOIP KO cell lysates transfected with luciferase reporters and HOIP WT where indicated. Cells were treated for 24 h with L18-MDP (200 ng/ml) or TNF (10 ng/ml) as indicated. To the right, HCT-116 WT and HOIP KO cell lysates were analyzed by immunoblotting as indicated. Data in (A) and (C) represent the mean ±SEM of at least three independent experiments, each performed in duplicate. $^{**}p < 0.01$, *n.s.,* not significant.

Figure S3. CYLD catalytic activity inhibits the NOD2 pathway upstream of nuclear translocation of NF-κB. Data are associated with Figure 3.

(A) Data from Figure 3C plotted as mean fluorescence intensity (MFI) of all cells in the IL-8/APC channel. (B) Untransfected cells from the experiment shown in Figure 3C plotted as percentage of IL-8

positive cells and MFI for all cells in the IL-8/APC channel. (C) and (D) HEK293FT cell lysates from experiments shown in Figure 3D (C), Figure 3E (D) were analyzed by immunoblotting as indicated (F) Schematic depiction of the engineered AVPI-Ub4GS protein binding to XIAP BIR2 and BIR3 domains via an N-terminal IBM to activate NF-κB. (F) NF-κB activity in HEK293FT cell lysates transfected with dual luciferase reporters and vector, inactive $XIAP^{F/A}$, AVPI-Ub4GS, CYLD or OTULIN as indicated, below HEK293FT cell lysates were analyzed by immunoblotting as indicated.

Data in (A), (B) and (F) represent the mean ±SEM of at least three independent experiments, each performed in duplicate (D-F). $^{**}p < 0.01$, *n.s.,* not significant.

Figure S4. CYLD limits extension of Ub chains on RIPK proteins. Data are associated with Figure 4.

(A) Endogenous Ub conjugates from Figure 4A purified using biotin-K63-SUB from U2OS/NOD2 cells and cell lysates at the indicated time points after treatment with L18-MDP (200 ng/ml).

Figure S5. Inhibition of IAPs reveals extensive regulation of RIPK2 ubiquitination by CYLD and OTULIN. Data are associated with Figure 5.

(A) and (B) Purified Ub conjugates using TUBE or UBA^{Ubq} as indicated from U2OS/NOD2 cells from the experiment shown in Figure 5A (A) and Figure 5B (B) were analyzed by immunoblotting against Ub.

Figure S6. CYLD trims Lys63- and Met1-Ub on RIPK2. Data are associated with Figure 6.

(A-C) UbiCRest analysis of ubiquitinated RIPK2 isolated with UBA^{Ubq} from L18-MDP treated U2OS/NOD2 (A and B) and THP-1 (C) cells (200 ng/ml, 1 h). Purified Ub conjugates were incubated with the indicated deubiquitinases with or without a spike in of GST-Met1-Ub4 for the indicated time at 30°C, and samples were examined by immunoblotting of RIPK2 or antibodies that recognize all Ub linkages, Lys63-Ub or Met1-Ub. The anti-RIPK2 blots in (A) are from the same gel but have been cut in two and tilted to adjust for bending of the gel. Asterisk in (B) denotes cross-reactivity of the RIPK2 antibody with recombinant GST-Met1-Ub4 added to the reaction.

Figure S7. Lys63-Ub and Met1-Ub are individually indispensable for NOD2 signaling. Data are associated with Figure 7.

(A) Mean fluorescence intensity of all U2OS/NOD2 cells in the GFP channel transfected with GFP, GFP-K63-SUB or GFP-M1-SUB with or without the nuclear localization signal. The cells were either left untreated or stimulated with L18-MDP (200 ng/ml, 4 h). (B) Data from Figure 7D plotted as mean fluorescence intensity (MFI) of all cells in the IL-8/APC channel. Data represent the mean ±SEM of at least three independent experiments, each performed in duplicate. $^{**}p < 0.01$, $^{***}p < 0.001$.

Supplemental References

Gyrd-Hansen, M., Darding, M., Miasari, M., Santoro, M.M., Zender, L., Xue, W., Tenev, T., da Fonseca, P.C., Zvelebil, M., Bujnicki, J.M.*, et al.* (2008). IAPs contain an evolutionarily conserved ubiquitin-binding domain that regulates NF-kappaB as well as cell survival and oncogenesis. Nat Cell Biol *10*, 1309-1317.

Watabe-Uchida, M., John, K.A., Janas, J.A., Newey, S.E., and Van Aelst, L. (2006). The Rac activator DOCK7 regulates neuronal polarity through local phosphorylation of stathmin/Op18. Neuron *51*, 727-739.

Figure S1

A
for Figure 1D

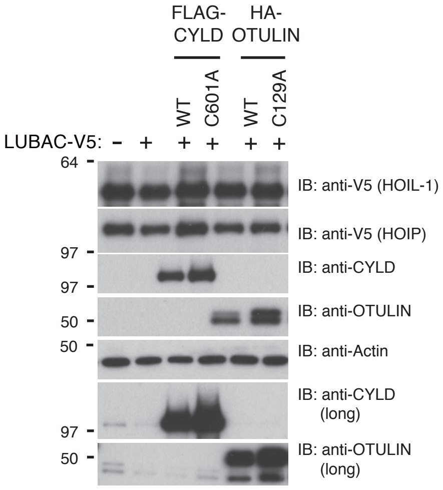

B
For Figure 1D and 1H

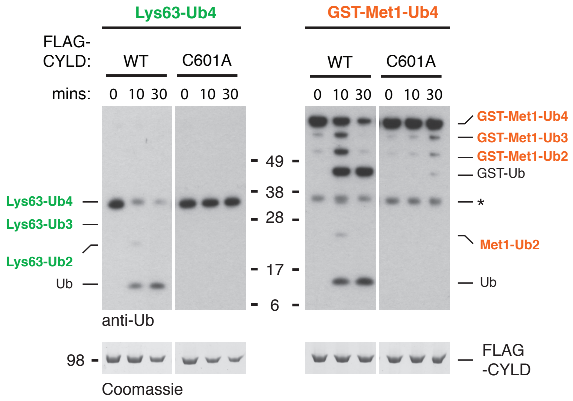

C
for Figure 1E

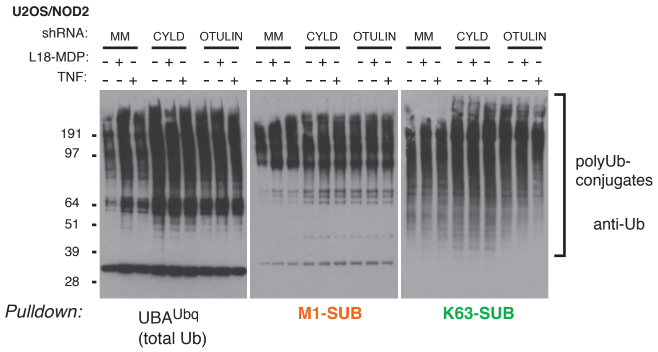

D
for Figure 1F

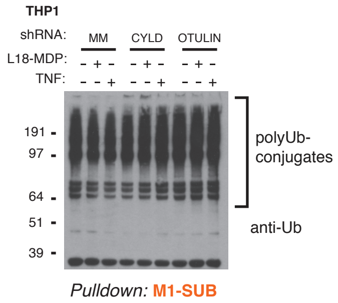

E
for Figure 1G

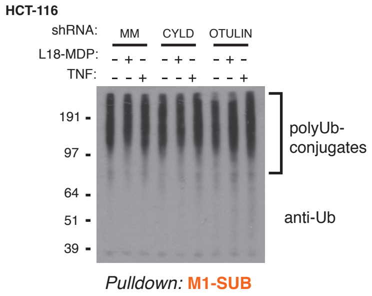

F
for Figure 1H

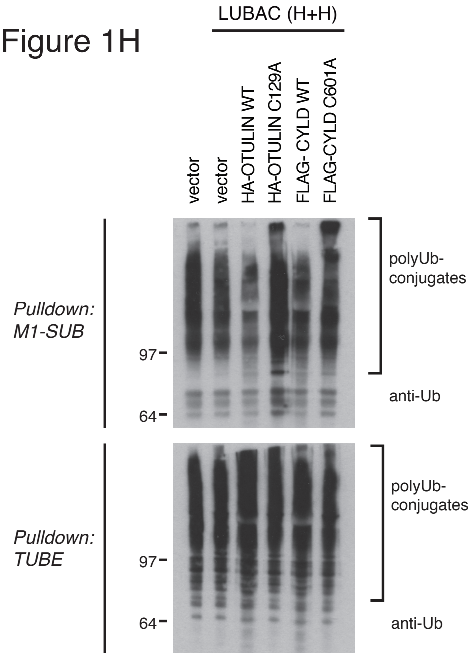

Figure S2

A
for Figure 2D

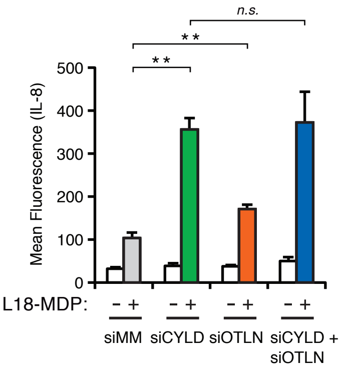

B
for Figure 2D

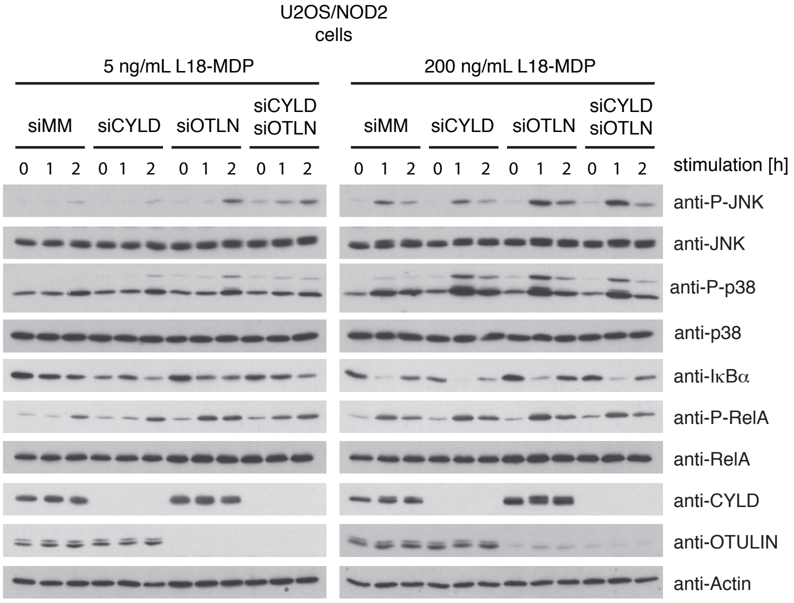

C
for Figure 2F

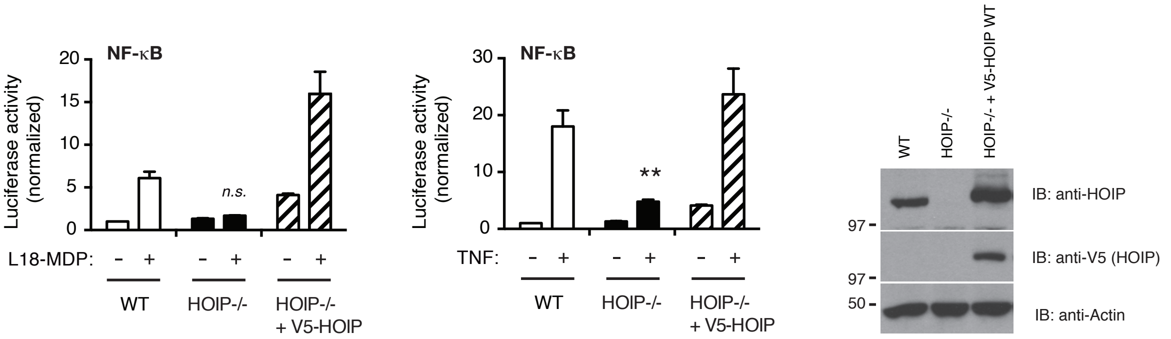

Figure S3

A
for Figure 3C

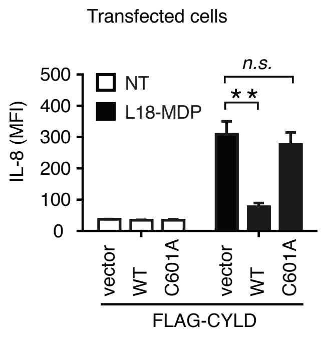

B
for Figure 3C

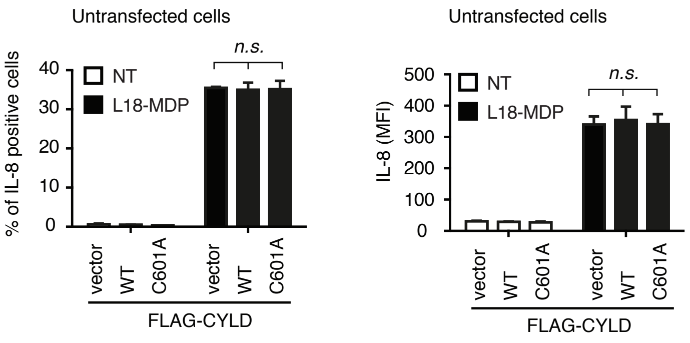

C
for Figure 3D

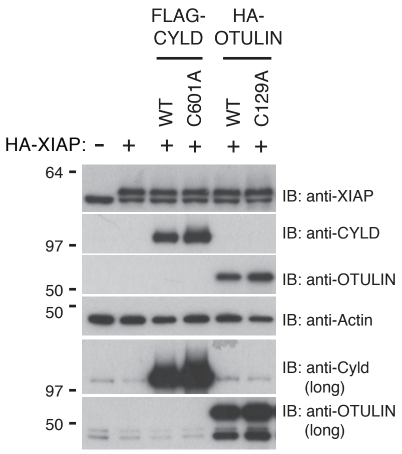

E
for Figure 3D

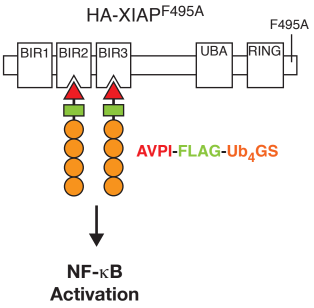

F
for Figure 3D

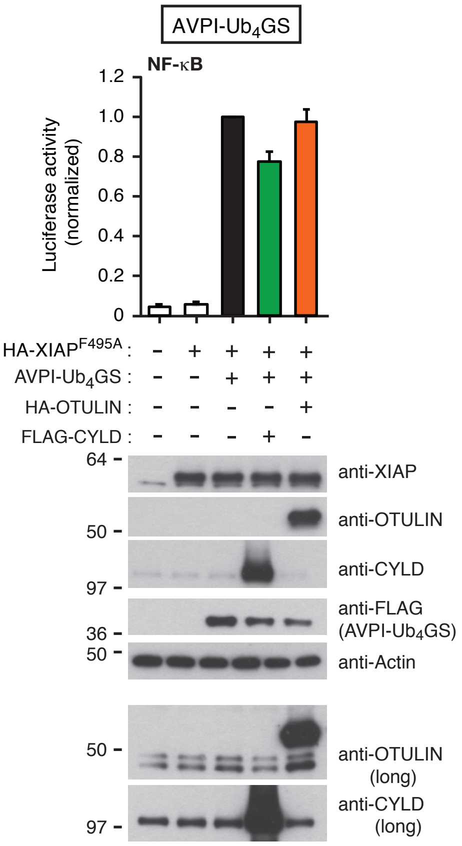

D
for Figure 3E

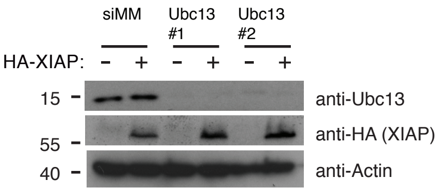

Figure S4

A
for Figure 4A

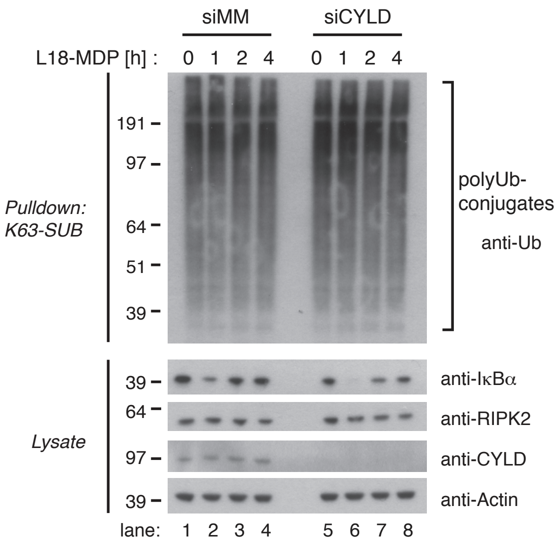

Figure S5

A
for Figure 5A

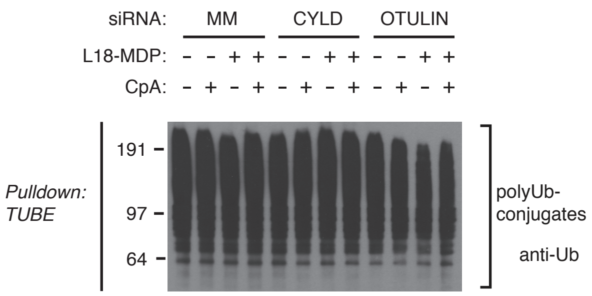

B
for Figure 5B

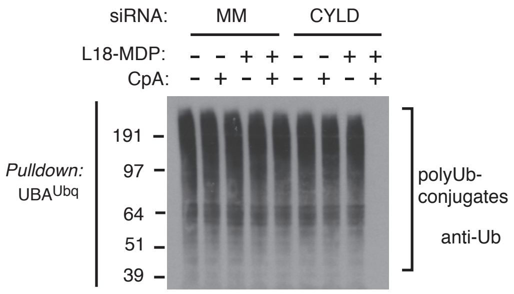

Figure S6

A
for Figure 6B

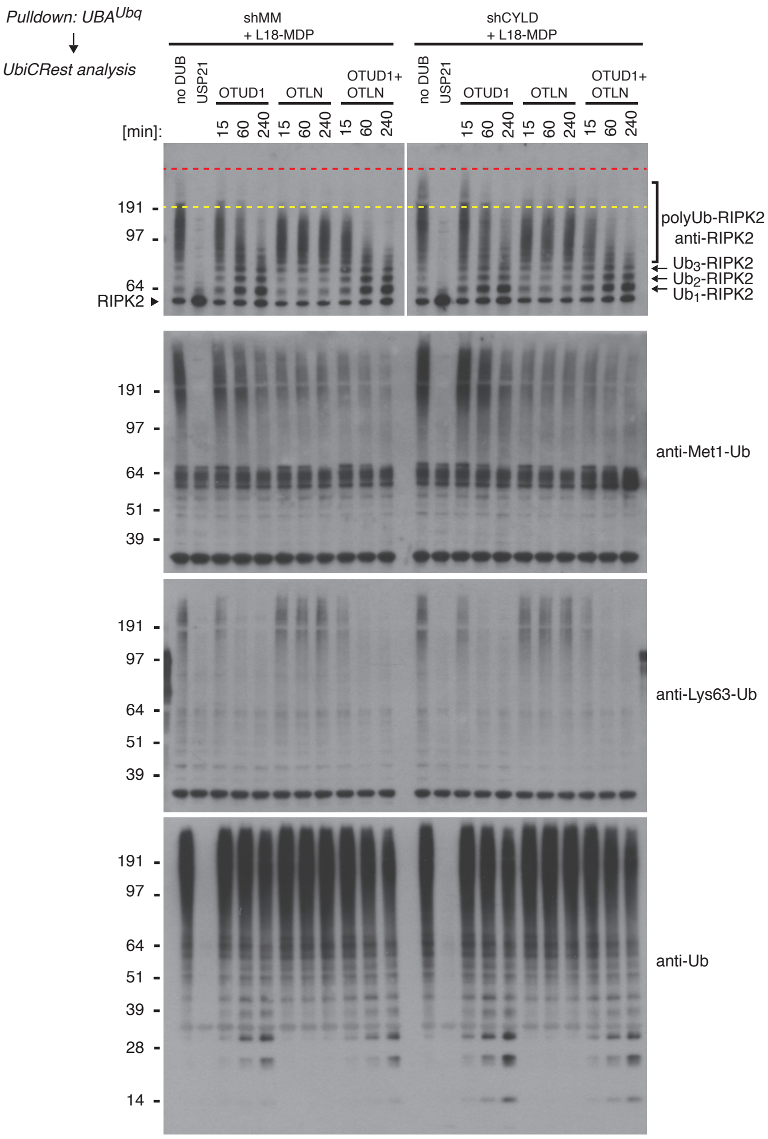

B
for Figure 6B

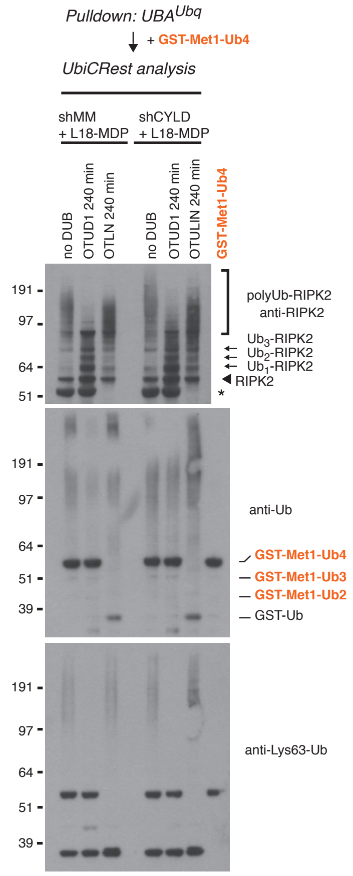

C
for Figure 6C

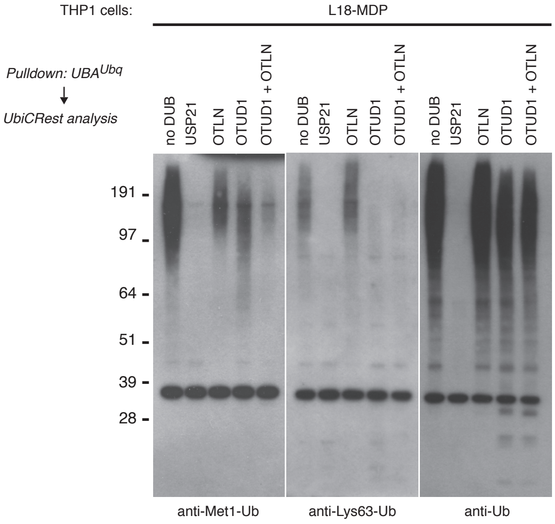

Figure S7

A

for Figure 7D and 7F

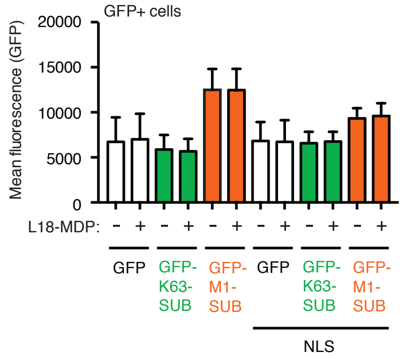

B

for Figure 7D

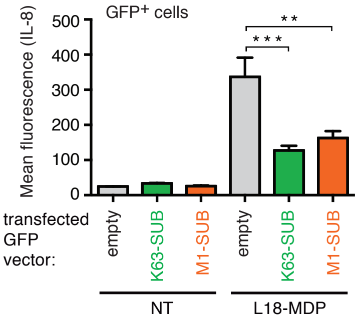

Supplement: Document S2. Article plus Supplemental Information [file mmc2.pdf]
